# Peripheral blood T-cell signatures from high-resolution immune phenotyping of γδ and αβ T-cells in younger and older subjects in the Berlin Aging Study II

**DOI:** 10.1186/s12979-015-0052-x

**Published:** 2015-12-04

**Authors:** Kilian Wistuba-Hamprecht, Karin Haehnel, Nicole Janssen, Ilja Demuth, Graham Pawelec

**Affiliations:** Department of Internal Medicine II, University Medical Center, Waldhörnlestr. 22, Tübingen, 72072 Germany; Department of Dermatology, University Medical Center, Tübingen, Germany; Research Group on Geriatrics, Charité - Universitaetsmedizin, Berlin, Germany; Institute of Medical and Human Genetics, Charité-Universitätsmedizin Berlin, Berlin, Germany; The John van Geest Cancer Research Centre, School of Science and Technology, Nottingham Trent University, Clifton Lane, Nottingham, NG11 8NS UK

**Keywords:** γδ T-cells, αβ T-cells, CMV, Aging, Senescence, Differentiation Phenotypes, Flow Cytometry

## Abstract

**Background:**

Aging and latent infection with Cytomegalovirus (CMV) are thought to be major factors driving the immune system towards immunosenescence, primarily characterized by reduced amounts of naïve T-cells and increased memory T-cells, potentially associated with higher morbidity and mortality. The composition of both major compartments, γδ as well as αβ T-cells, is altered by age and CMV, but detailed knowledge of changes to the γδ subset is currently limited.

**Results:**

Here, we have surveyed a population of 73 younger (23–35 years) and 144 older (62–85 years) individuals drawn from the Berlin Aging Study II, investigating the distribution of detailed differentiation phenotypes of both γδ and αβ T-cells. Correlation of frequencies and absolute counts of the identified phenotypes with age and the presence of CMV revealed a lower abundance of Vδ2-positive and a higher amount of Vδ1-positive cells. We found higher frequencies of late-differentiated and lower frequencies of early-differentiated cells in the Vδ1+ and Vδ1-Vδ2-, but not in the Vδ2+ populations in elderly CMV-seropositive individuals confirming the association of these Vδ2-negative cells with CMV-immunosurveillance. We identified the highest Vδ1:Vδ2 ratios in the CMV-seropositive elderly. The observed increased CD4:CD8 ratios in the elderly were significantly lower in CMV-seropositive individuals, who also possessed a lower naïve and a larger late-differentiated compartment of CD8+ αβ T-cells, reflecting the consensus in the literature.

**Conclusions:**

Our findings illustrate in detail the strong influence of CMV on the abundance and differentiation pattern of γδ T-cells as well as αβ T-cells in older and younger people. Mechanisms responsible for the phenotypic alterations in the γδ T-cell compartment, associated both with the presence of CMV and with age require further clarification.

**Electronic supplementary material:**

The online version of this article (doi:10.1186/s12979-015-0052-x) contains supplementary material, which is available to authorized users.

## Background

Aging is accompanied by a dysregulation of the immune response with implications for health [1]. Developmentally-programmed thymic involution causing reduced release of naïve T-cells in adults results in the characteristic accumulation of memory T-cells and reduction of naïve T-cells over the lifecourse [2]. Protection against new infections is impaired due to a reduced naïve T-cell repertoire, and control of previously-encountered pathogens may be impaired by senescence of the memory cells. Thus, accumulation of memory T-cells and reduction of naïve T-cells are commonly taken as hallmarks of immunosenescence, although they mostly reflect adaptive responses [3]. Similar shifts in proportions of memory T-cells are seen as a result of infection with Cytomegalovirus (CMV) [4], suggesting the presence of the latter as one of the major factors contributing to this phenomenon. Infection with this widespread β-herpesvirus is usually asymptomatic, and establishes occult latency. Nonetheless, primary infections or re-infections with this virus can be life-threatening for immunocompromised people or newborns, indicating that CMV is a powerful pathogen requiring immune control. Infected individuals possess serum antibodies specific for CMV and are thus referred to as CMV-seropositive. The majority of infected people present with expanded memory phenotype CD8+ T-cell populations, and may have a higher risk of coronary heart disease associated with vascular inflammation [5, 6] or diabetes [7]. Seroprevalence depends on age and socio-economic factors. A study of 24,260 Germans yielded a seroprevalence of 46 % in the age range 18–60 years with a yearly conversion rate of 0.55 % (http://www.rki.de/DE/Content/Infekt/EpidBull/Merkblaetter/Ratgeber_Zytomegalievirus.html). Hence, there is a chance of becoming infected with CMV at any time of life, and the proportion of the population that is infected thus increases with age.

Surveys of T-cell biomarkers for immune monitoring purposes commonly focus on the most prominent T-cell subset, expressing T cell receptors (TCR) for antigen composed of αβ chains and mostly either CD4 or CD8 co-receptors. Age-associated as well as CMV-associated differences are well-recognized in both subsets, but more markedly in the CD8+ subset [1–4]. Lower frequencies of CD8+ naïve T-cells and higher proportions and absolute numbers of late-stage differentiated CD8+ T-cells expressing CD45RA (sometimes designated “TEMRA” cells) are commonly taken as key-markers of immune aging [4]. In the Swedish OCTO-study of people 85 years old at baseline, an inverted CD4:CD8 ratio of <1 resulting from an accumulation of large numbers of CD8+ TEMRA cells was associated with poorer survival at 2-, 4- and 6–year follow-up [8]. At the other extreme, the Belgian BELFRAIL study associated a CD4:CD8 ratio >5 resulting from large numbers of naïve CD4+ T-cells with poorer health and more frailty at follow-up [9] and with worse 3-year survival in women (Adriaensen et al., manuscript in preparation).

Other studies in different cohorts are also examining the influence of these αβ T-cell-based variables on health and survival in the elderly. However, in addition to the well-described αβ T-cells, a second discrete subset of T-cells is present in the peripheral blood of all individuals. These cells express a completely different TCR composed of γδ not αβ chains, and which are mostly CD4- and CD8-double negative (with a minor CD8+ population [10]). Limited TCR polymorphism, especially in the δ chains, and lack of MHC-restriction for antigen recognition position these cells somewhere between innate and adaptive immunity [11]. In healthy adults 1–10 % of all peripheral T-cells carry this TCR. Analogous to the presentation of peptides via MHC molecules to the αβ TCR, glycolipids are presented via CD1 to γδ T-cells [12, 13]. The majority of the γδ T-cells in peripheral blood express the δ2 chain, a minority the δ1 chain [14], and other much smaller populations express other δ-chains. Those γδ T-cells expressing the Vδ2 isoform recognize small phosphoantigens derived from the non-mevalonate pathway or the isoprenoid biosynthesis [15, 16] (stress, pathogen or tumor-associated), alkylamines [17] or synthetic aminobisphosphonates [18, 19]. In contrast, Vδ1 cells recognize stress-induced ligands such as MICA, MICB [20], or EPCR [21]. In the context of aging, there are few studies on γδ T-cells reporting that their abundance in peripheral blood is reduced in the elderly [22, 23]. A considerable increase of γδ T-cells during active CMV infection has been reported [24], which potentially associates these cells with anti-CMV immune responses, as shown by many others [25–27]. As the proportion of the population infected with CMV increases with age, this could counteract the age-associated decrease. The purpose of the present study was to survey a younger and older population to seek age-associated differences in peripheral γδ T-cells, taking this effect of CMV infection into account. Moreover, we have analyzed the main γδ T-cell compartments (Vδ1+, Vδ2+ and Vδ1-Vδ2-) separately because CMV infection and aging result jointly in an increased pool of Vδ2-negative cells [23, 26, 28–30] with altered memory phenotype distributions [31, 32]. We previously showed an association of the Vδ2-negative cells with anti-CMV IgG-titers [32]. This may be related to the antibody-dependent anti-CMV activity of Vδ2-negative γδ T-cells dependent on their CD16 expression [29] and their recognition of other stress-induced molecules [33]. We therefore included CD16 in the present phenotypic analysis focused particularly on γδ T-cells in a younger and older population drawn from the Berlin Aging study II (BASE-II) to determine age- and CMV-associated alterations at the cellular level. These results emphasize the impact of CMV infection on most αβ and γδ T-cell subsets, including the rarely-studied CD8+ γδ T-cells.

## Results

### T-cell phenotypes in the context of age and CMV

**Fig. 1:**
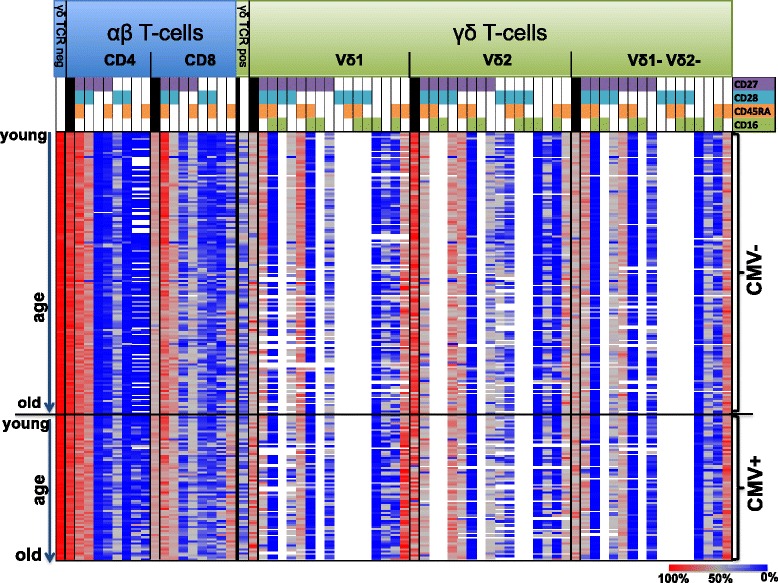
Detailed distribution of αβ T-cell and γδ T-cell compartments and differentiation phenotypes. Each line reflects frequencies of a single individual, color-coded. Red indicates high values, blue low. The first part of the heat map describes the CD4+ and CD8+ αβ T-cells (blue background) and their differentiation status displayed in columns and characterized by the expression of CD27 (purple), CD28 (turquoise), CD45RA (orange) and CD16 (green), whereas white indicates the absence of the individual markers. The second part of the heat map describes the abundance of the Vδ1+, Vδ2+ and Vδ1-Vδ2- γδ T-cell compartments and their differentiations signatures. The black lines in the coding-table for the differentiation markers show the frequencies of the parental compartments. The heat map is horizontally divided into CMV-seronegatives (CMV-) in the upper and seropositives (CMV+) in the lower part. Both parts are further stratified for subject age

Large scale surveys of the proportions and differentiation phenotypes of γδ T-cells in cross-sectional studies of younger and older populations are scarce. Taking advantage of the Berlin BASE-II study, here we have undertaken detailed phenotyping of peripheral γδ T-cells in 73 younger and 144 older individuals, in relation to their CMV-serostatus. At the same time, we assessed similar parameters for the αβ T-cell subset in comparison in order to confirm the expected normalcy of this population with regard to established T-cell biomarkers. Using advanced flow cytometry and the standardized OMIP-20 panel specifically designed for accurately determining γδ T-cell phenotypes [34], we analyzed a total of 217 individuals. These data are summarized in Fig. 1, which displays the distribution of all major memory-differentiation stages (vertical columns), for each individual tested (horizontal lines). The frequencies of each phenotype are color-coded (high being red, low blue and white absent). The upper part of the figure depicts the effect of age on CMV-seronegative individuals, with age increasing from top to bottom, whereas the lower part of the figure shows the same for CMV-seropositive subjects. Dominant effects of CMV-seropositivity are observed in terms of higher total frequencies of the Vδ1+ compartment and lower frequencies in the Vδ2+ compartment, whereas lower proportions of the CD8+ compartment reveal jointly the effects of the factors age and CMV-seropositivity as influences on their abundance (Fig. 1). Notably, there are higher frequencies of the late-differentiated phenotypes (CD27-CD28-CD45RA + CD16-) in the CD8+ and Vδ1+ T-cells of CMV-seropositive individuals. As the white-colored areas indicate, the expression of CD16 in the γδ T-cell compartment is limited only to some very early- and very late-differentiated memory subsets. The following sections will describe differences in phenotypic abundance and absolute cell counts in greater detail.

### Composition of the T-cell compartments is associated with age and CMV-serostatus

The highest proportions of γδ T-cells were found in young CMV-seronegative individuals, at a median frequency of 3.8 % of all CD3+ T-cells and a median absolute count of 51 cells/μL blood. This was significantly different in older individuals, independent of their CMV-serostatus (first lines of each section comparing young seronegative with old seropositive and seronegative individuals in Additional file 1: Table S1: *p* = 0.0052, *p* < 0.0001 respectively and Additional file 1: Table S4: *p* = 0.0006, *p* < 0.0001 respectively). For reference purposes, additional tables (Additional file) show in detail the p values of the Mann-Whitney comparisons of the 4 groups and the median frequency/counts of the latter on all identified cellular populations.

**Fig. 2:**
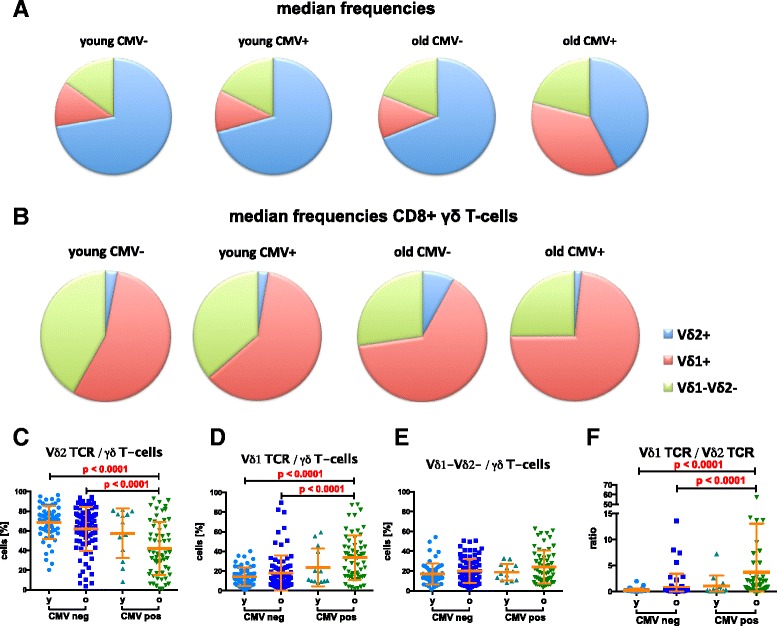
Phenotypic distribution of the Vδ1+, Vδ2+ and Vδ1-Vδ2- γδ T-cell subsets in young and old CMV seropositive and seronegative individuals. (**a**) Median frequencies of the three subsets in the total γδ T-cells and (**b**) in the CD8+ group of γδ T-cells. Detailed differences are shown between young (y) and old (o) CMV-seropositives and seronegatives of Vδ2+ cells (**c**), Vδ1+ cells (**d**), Vδ1-Vδ2- cells (**e**) and the Vδ1:Vδ2 ratio (**f**). The Mann–Whitney test was used for the statistical comparison. Bonferroni-correction adjusted the significance cutoff to p ≤ 0.0083

γδ T-cells in peripheral blood were classified into Vδ2+, Vδ1+ or the pool of other γδ T-cells carrying neither (Vδ1-Vδ2-). Most γδ T-cells are predominantly Vδ2+ in younger subjects, independent of their CMV-serostatus. The same is true in older CMV-seronegatives but not in CMV-seropositives (blue sections in Fig. 2a and Additional file 1: Figure S1). The latter have a nearly equal proportion of Vδ2+ and Vδ1+ cells (37.9 and 32.9 %, or median values of 10 and 8 cells/μL blood, respectively). Fig. 2c displays a gradual reduction of the median frequencies of the Vδ2+ compartment starting with young CMV-seronegative with the highest frequencies, then old CMV-seronegatives, young CMV-seropositives and finally the older CMV-seropositive subjects who have the lowest frequencies. There is a reciprocal increase of the Vδ1+ compartment (Fig. 2d; for statistical evaluation, see Additional file 1: Table S1). As a group, young and old CMV-seronegatives were not significantly different from one another in this respect, although some of the older individuals had much higher frequencies of this cell type. Statistical significance was achieved, however, for the comparison of the frequencies in old and young CMV-seronegatives vs old seropositives showing CMV as an enhancing factor of age-associated alterations (Fig. 2c: *p* < 0.0001 for both).

Similar patterns were identified when analyzing absolute cell counts (Additional file 1: Table S4), but statistical evaluation revealed a slightly different scenario: Lower counts of Vδ2+ cells were found in the old, regardless of CMV-serostatus compared to young seronegative (Additional file 1: Table S4). Young subjects, regardless of their CMV-serostatus, have more Vδ1+ cells than old CMV-seronegatives (Additional file 1: Table S4) whereas old CMV-seropositives have the highest count of all and significantly higher counts than old seronegative individuals (Additional file 1: Table S4, *p* < 0.0001). Relative frequencies of the double-negative Vδ1-Vδ2- compartment did not differ significantly (Fig. 2e), although we did find higher absolute counts in the young CMV-seronegative and old CMV-seropositive compared to old CMV-seronegative subjects (Additional file 1: Table S4). Significantly higher ratios were observed in CMV-seropositive old compared to old or young seronegative subjects (Fig. 2f and Additional file 1: Table S1 and S4, *p* < 0.0001 for all).

About 4.9–10 % of all γδ T-cells express CD8 on the surface. We observed a higher frequency of these in old CMV-seropositive individuals, compared to old or young CMV-seronegatives (Additional file 1: Table S2, *p* = 0.0008 and *p* = 0.0002, respectively). In contrast to the entire γδ T-cell compartment, the majority of CD8+ γδ T-cells express the Vδ1 TCR (Fig. 2b). In the elderly there were significantly higher frequencies of CD8 + Vδ2+ cells in CMV-seronegatives than in seropositives (Additional file 1: Table S2, *p* = 0.0026). A shift in the proportions when comparing young CMV-seronegative with young CMV-seropositive, old CMV-seronegative or old CMV-seropositive individuals points towards a higher CD8 + Vδ1+ compartment as identified for the total Vδ1+ cells (Fig. 2b vs Additional file 1: Table S2). Highest proportions of the Vδ1-δV2- pool where identified in young CMV-seronegatives, compared to old, independent of the latter’s CMV-serostatus (Fig. 2b; Additional file 1: Table S2, *p* = 0.0002 comparing old and young seronegatives and *p* = 0.0005, old seropositives with young seronegatives).

**Fig. 3:**
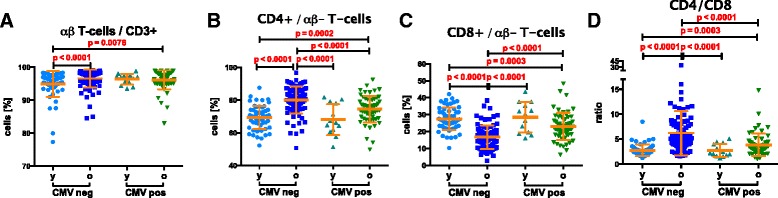
Age and CMV associated differences in the αβ T-cell compartment. (**a**) Frequencies of αβ T-cells, identified as γδ TCR negative, in young (y) and old (o) CMV seropositive and seronegative individuals. (**b**) The abundance of CD4+ and (**c**) of CD8 T-cells. (**d**) CD4:CD8 ratios. The Mann–Whitney test was used for the statistical comparison. Bonferroni-correction adjusted the significance cutoff to p ≤ 0.0083

Turning to the αβ T-cell subset, young CMV-seronegative subjects have a lower abundance of these compared to the old, independent of the latter’s CMV-serostatus (*p* = 0.0076 and *p* < 0.0001, respectively, Fig. 3a and Additional file 1: Table S4). We observed higher frequencies of CD4+ T-cells in the elderly than in the young, regardless of their CMV-serostatus (Fig. 3b) and reciprocally lower frequencies of CD8+ T-cells in the elderly, again independent of CMV serostatus (Fig. 3c). However, the CMV+ elderly have significantly lower frequencies of CD4+ T-cells than the CMV-negative elderly (*p* < 0.0001, Fig. 3b) and reciprocally, the CMV+ elderly have significantly higher frequencies of CD8+ T-cells than the CMV-negative elderly (Fig. 3c, *p* < 0.0001). The same pattern of significant differences was found for absolute counts in the CD8+ T-cell subset (Additional file 1: Table S4). However, no significant differences were observed for absolute CD4+ T-cell counts (Additional file 1: Table S4), consistent with the known greater effect of CMV on CD8+ than CD4+ T-cells. Accordingly, the CD4:CD8 ratio calculated on the basis of either counts or frequencies was greatly increased in old compared to young subjects, but interestingly this was only statistically significant in CMV-seronegative subjects (Fig. 3d, *p* < 0.0001). Old CMV-seropositives had lower values than old seronegatives (Fig. 3d, *p* < 0.0001) but higher than young CMV-seronegative individuals (Fig. 3d, *p* = 0.0003). Finally, young CMV-seropositives had lower numbers than old CMV-seronegative subjects (Fig. 3d, *p* < 0.0001).

### Memory cell subsets vary in abundance within the T-cell classes and phenotypes, showing different associations with age and CMV

**Fig. 4:**
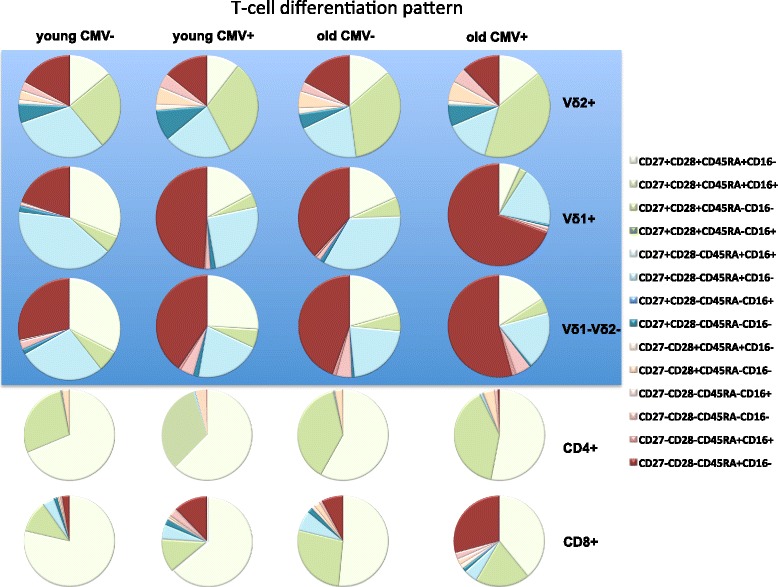
Differentiation-scheme of the entire identified γδ T-cell and αβ T-cell compartments. Each cellular compartment (lines) is represented by a pie-chart for young and old CMV-seropositive (CMV+) and seronegative (CMV-) individuals. The γδ T-cell compartment is shown with a blue background, the αβ T-cells with a white background. The single differentiation statuses are color coded in the pie charts, using green for early- and red for late-differentiated phenotypes

Memory cell subsets were identified by the presence or absence of CD27, CD28 and CD45RA. CD27 + CD28 + CD45RA+ T-cells are considered to be early-differentiated, whereas CD27-CD28-CD45RA+ cells are very late-differentiated. In addition, each memory cell subset in the γδ T-cell compartment was analyzed for expression of the F_c_γ III Receptor (CD16). γδ T-cells that have CD16 on their surface were previously described to be in involved in antibody-dependent anti-CMV immunity in a γδ TCR-independent manner [29]. We found that the diversity of memory phenotypes in the γδ T-cell compartments is similar to the CD8+ αβ T-cells, unlike in the CD4+ αβ T-cell subset (see overview displayed in Fig. 4). The latter consisted mainly of early-differentiated phenotypes in the elderly as well as the young, and with only slightly more differentiated cells even in CMV-seropositive elderly (Fig. 4). Within the γδ T-cell compartment, the Vδ2+ cells mainly showed an earlier-differentiated phenotype, in contrast to the Vδ1+ cells or the pool of the other (Vδ1-Vδ2-) γδ T-cells that revealed high proportions of later-differentiated cells (Fig. 4, upper panels). Analysis stratifying subjects according to CMV or age did not reveal any significant differences in the Vδ2+ compartment, neither for early- (CD27 + CD28 + CD45RA + CD16-) nor late-differentiated (CD27-CD28-CD45RA + CD16-) subsets (Additional file 1: Table S1). However, we observed lower frequencies of CD27 + CD28 + CD45RA-CD16- cells in young CMV-seronegative compared to old CMV-seropositive subjects (Additional file 1: Table S1 and Figure S2: *p* = 0.0004). Regardless of the age and independent of CD16 expression, CMV-seropositives had significantly lower proportions of CD27 + CD28-CD45RA+ cells compared to young seronegative individuals, indicating an association with the presence of CMV (Additional file 1: Table S1 and Figure S2). A clear age-associated difference was only observed when comparing CD27 + CD28-CD45RA + CD16- cells in young and old CMV-seronegative subjects (Additional file 1: Table S1 and Figure S2, *p* < 0.0001).

The main observations in the Vδ1+ memory compartment were that there were lower proportions of early-differentiated (CD27 + CD28 + CD45RA + CD16-) and reciprocally higher proportions of late-differentiated (CD27-CD28-CD45RA + CD16-) cells in young CMV-seronegative compared to old, regardless of the CMV-status of the latter (Additional file 1: Table S1 and Figure S3, *p* < 0.0001 for all). The same was true for old CMV-seropositive compared to old seronegative individuals (Table S1, *p* = 0.0045 and *p* < 0.0001 respectively). Additionally, higher frequencies of late-differentiated cells even in the young CMV-seropositive subjects relative to seronegatives of the same age-group were found (Additional file 1: Table S1 and Figure S3, 0.0018) suggesting an accumulation of late-differentiated cells in CMV-seropositives. Significantly reduced frequencies were observed comparing young CMV-seronegatives with old seropositives for the minor compartments with the phenotypes CD27 + CD28 + CD45RA-CD16-, CD27 + CD28-CD45RA + CD16- and CD27 + CD28-CD45RA-CD16-. The same reduction was found for the latter phenotype comparing old CMV-seronegative and seropositive subjects. Furthermore, a greater abundance of the CD27 + CD28-CD45RA + CD16- phenotype was identified in young CMV-seronegative than in young or old seropositives. However, no statistically significant differences were identified between young and old CMV-seronegatives for the latter phenotype (Additional file 1: Table S1 and Figure S3). No CD27-CD28+ phenotypes were detectable, neither for the Vδ1+ nor the other γδ T-cells (Vδ1-Vδ2-) (Additional file 1: Figure S3 and S4).

The memory phenotype distribution of the pool of other γδ T-cells (Vδ1-Vδ2-) (Additional file 1: Figure S4) revealed patterns similar to the Vδ1+ compartment (Additional file 1: Figure S3). Again, significantly higher frequencies of the early-differentiated (CD27 + CD28 + CD45RA + CD16-) and reciprocally lower abundance of the late-differentiated compartments (CD27-CD28-; independent of the expression of CD45RA, and even identified on the CD45RA + CD16+ cells) were found comparing young CMV-seronegative with old subjects regardless of the latter’s serostatus (Additional file 1: Table S1). Lower frequencies were identified in the effector cell compartment of old seropositive subjects compared to young CMV-seronegative with individuals for the phenotypes CD27 + CD28 + CD45RA-CD16-, CD27 + CD28-CD45RA + CD16- and CD27 + CD28-CD45RA-CD16- (Additional file 1: Table S1, *p* = 0.0071, *p* = 0.0005, *p* < 0.0001 respectively). Significantly lower frequencies of the latter were observed as well when comparing young with old CMV-seropositives. Again, we identified lower frequencies of the CD27 + CD28-CD45RA + CD16- phenotype in old compared to young CMV-seronegatives (Additional file 1: Table S1).

For the discrimination of the CD8+ γδ T-cell memory phenotype only the expression of CD27 and CD28 was considered, as these cells were too low in abundance for further subdivision. There were then too few CD8+ Vδ2+ T-cells for an analysis of the memory phenotypes at all, but the CD8+ Vδ1+ compartment (Additional file 1: Figure S5 A and Table S2) revealed similar differentiation patterns compared to the total Vδ1+ γδ T-cells (Fig. 4 compared to Additional file 1: Figure S3 and Table S1), namely, significantly higher frequencies of early-differentiated (CD27 + CD28+) cells in young CMV-seronegatives compared to the old, regardless of their serostatus (Additional file 1: Table S2: *p* < 0.0001). Reciprocally, significantly higher frequencies of late-differentiated cells (CD27-CD28-) were seen when comparing old CMV-seropositive with seronegative individuals (in young and old) (Additional file 1: Figure S5 A and Table S2). The effector phenotype CD27 + CD28- was significantly higher in the young CMV-seronegatives than in the old individuals, regardless of their CMV-serostatus. Again a similar pattern was observed for the pool of the other CD8+ γδ T-cells (Vδ1-Vδ2-) (Additional file 1: Table S2 and Figure S5 B) compared to the same subset in all γδ T-cells (Fig. 4). Young CMV-seronegatives had higher proportions of early-differentiated cells than old CMV-seropositives or seronegatives, but comparing young and old seropositives showed that the latter have lower frequencies of those cells. Again, a higher abundance was found for the late-differentiated phenotype (CD27-CD28-) comparing both groups of older individuals with young CMV-seronegatives. Young CMV-seropositives had significantly higher frequencies of these cells than seronegatives of the same age, as also identified for the CD8 + Vδ1+ compartment (Additional file 1: Figure S5 A and B). The Vδ1-Vδ2- CD8+ compartment was the only one that showed higher frequencies of the rare phenotype CD27-CD28+ in the elderly, independent of the presence of CMV, compared to the young CMV-seronegative subjects.

CD8+ αβ T-cells revealed the most differences in memory phenotype distribution as expected (Fig. 4, lower panels). Lower frequencies of early-differentiated and higher frequencies of late-differentiated cells were found in older and CMV-seropositive subjects (Additional file 1: Figure S6 and Table S3: either trends or statistical significance was observed for all comparisons). A gradual CMV-dominated pattern was only identified comparing the late-differentiated phenotypes (CD27-CD28-CD16-, independent of the CD45RA-expression) whereas a joint influence of age and CMV was observed for the early-differentiated cells (CD27 + CD28 + CD45RA + CD16-) (Additional file 1: Figure S6). Highest median frequencies were identified in the effector cell compartments in older CMV-seronegative individuals, suggesting CMV as a driving force towards accumulated late-differentiated and a diminished effector cell compartment in the elderly CMV-seropositive individuals. Statistical analyses, displayed in Additional file 1: Table S3, indicate that in addition to the major increase of the late-differentiated CD8+ compartment, memory phenotypes of the CD4+ cells do show similar patterns at a lower level. Interestingly, the small, very late-differentiated CD4+ subset (CD27-CD28-CD45RA + CD16-) was present essentially only in old CMV-seropositive compared to CMV-seronegative subjects (Additional file 1: Table S3, *p* < 0.0001 for both and Additional file 1: Figure S7). Similar to the CD8+ compartments of less-differentiated cells, no gradual patterns for the CD4+ compartment were identified with the exception of the CD27-CD28-CD45RA-16- cells (Additional file 1: Figure S7). Age-dependent effects seem to dominate this compartment of T-cells with a large early differentiated/effector cell compartment.

## Discussion

We present in this study a comprehensive and highly detailed analysis of the whole peripheral blood T-cell compartment in 73 younger and 144 older individuals drawn from the BASE-II study [35]. The present paper reports the results of an analysis of one-tenth of the total BASE-II cohort, already a large population to be subjected to this level of detailed immune cell phenotyping. We confirm the generally-acknowledged robust effects of age and CMV infection on the abundance and memory phenotype distribution of many T-cell compartments with an emphasis on the less well-studied γδ T-cell subsets. For this purpose, the advanced, well standardized and established flow cytometry panel, published as OMIP-20 [34], was employed.

### T-cell subsets

There are many reports describing differences in the αβ T-cells in younger and older individuals. Here, we report that aging is associated with a higher abundance of CD4+ and less CD8+ αβ T-cells. In the elderly, CMV-seropositive subjects possessed a smaller CD4+ and a larger CD8+ compartment compared to seronegative individuals. Thus, we confirm the presence of a latent CMV-infection as a factor that alters the αβ T-cell distribution towards a signature that is described in young subjects. The CD4:CD8 ratio reflects these findings: a significantly lower ratio was found in CMV-seropositive than seronegative elderly, although the latter still had a higher ratio than young CMV-seronegatives. This illustrates the independent effects of age and CMV infection, suggesting a potentially positive effect of CMV in our elderly cohort. Interestingly, Adriaensen at al. [9] reported recently that a CD4:CD8 ratio >5 was only present in the elderly in the BELFRAIL study, never in the young, caused by a shrinking CD8+ compartment. This phenotype was naïve T-cell dominated, with less late-differentiated CD8+ T-cells, lower CMV-specific IgG titers and worse physical condition [9], as well as poorer 3-year survival (manuscript in preparation). These intriguing data are consistent with a requirement for vigorous CMV-specific immunosurveillance to ensure good health and survival in later life, as suggested by results from our earlier study on the Leiden 85-Plus population [36]. This is also consistent with later follow-up studies from the Swedish NONA-study where none of those individuals who survived to become nonagenarians and centenarians had an inverted CD4:CD8 ratio, suggesting selection against individuals with this characteristic [37].

The other main T-cell compartment, the γδ T-cell population, is underrepresented in investigations regarding associations with aging and the chronic stimulation through persistent CMV infections, and this was the main focus of our present report. In infections, γδ T-cells seem to respond earlier than αβ T-cells, suggesting that they are part of the “first line of defense” and the initiation of an inflammatory response. Consistent with this notion, γδ T-cells are potent producers of pro-inflammatory cytokines like IL-17, IFNγ and TNF [38]. There are several studies associating certain sub-groups of γδ T-cells with anti-CMV immunity [24, 25, 33]. Previous observations report a reduction of the total γδ T-cell compartment that is associated with chronological aging [39], whereas a primary CMV infection causes a marked increase of these cells [24]. This observation is similar to what is seen in the CD8+ αβ T-cell compartment, which is also reduced with chronological aging, but can be increased through induction of clonal expansion by pathogens [4]. Several studies show that Vδ2-negative γδ T-cells correlate with both aging and latent CMV infection [31, 32]. Interestingly, many of the studied γδ T-cell clones possessing reactivity against CMV-infected cells as also show reactivity against transformed cells (for example [27]). This observation suggests the recognition of an ensemble of endogenous molecules that are upregulated in both responses to infections and cellular dysregulation [38].

The Vδ2-negative subset can be subdivided into the Vδ1+ compartment and the pool of others (Vδ1-Vδ2-), as we did in this study. We show that the frequency in the elderly of the Vδ1+ T-cells and not the pool of other γδ T-cells (Vδ1-Vδ2-) is markedly affected by CMV-seropositivity, although this is not the case for absolute cell counts. In contrast the hypothesis of a beneficial dual-reactivity of γδ T-cells (against transformed and CMV-infected cells) [40], we reported recently a negative association of the Vδ1+ compartment with the overall survival of late-stage melanoma patients [41].

The Vδ2+ compartment is reported to be lower in the elderly than the young [31]. These cells are associated mostly with pathogen challenges, infectious diseases and even tumor-induced stress via for example phosphoantigens of the non-/mevalonate pathway [42, 43]. The present study demonstrates that the gradual reduction of the Vδ2+ cells is not necessarily due to an gradual increase of the Vδ1+ compartment (significance was not achieved for all cases) as shown by examining the three different sub-groups of γδ T-cells. This observation is similar to what is described for the balance between CD4+ and CD8+ αβ T-cells. The fact that there is a direct correlation between the two prominent γδ T-cell subsets, age and CMV-seropositivity on the single subject level is shown through the alteration of the Vδ1:Vδ2 ratio. We show that this ratio is significantly increased in elderly CMV-seropositive individuals. Increased values for this ratio are also found in melanoma patients [41, 44], suggesting a potentially prominent role of these cells in triggering immune responses.

The presence or absence of CD8 is another confounding factor; while most likely not needed for activation through the γδ TCR, it defines a sub-group of γδ T-cells that mostly express the δ1 isoform and others (Vδ1-Vδ2-) of the γδ TCR. Further investigation of the meaning and functional capacity of this γδ T-cell is required.

### Memory phenotypes of the different T-cell subsets

The abundance of the identified memory phenotypes of the αβ T-cell compartment confirms the common consensus. Reduced proportions of naïve CD8+ cells were found in the elderly. Higher frequencies of late-differentiated cells (CD27-CD28-CD45RA + CD16-) mostly in the CD8+ compartment were identified in the old subjects, with the greatest difference found in the old CMV-seropositives compared to all other groups, as reported previously [4, 45–47]. High proportions of these late-differentiated CD8+ memory cells are reported to recognize CMV associated antigens, but not antigens of other persistent herpesviruses [48].

Our findings in the CD4+ compartment show, besides slight reduction of proportions of the early-differentiated cells, higher proportions of late differentiated cells (CD27-CD28-CD45RA + CD16-) in old CMV-seropositive subjects. This population was clearly identifiable and confirms the findings earlier reported on T-cell distribution in the BASE-II study [49] although the existence of this subset is controversial. This accumulation of CD4+ TEMRA cells in old CMV-seropositive individuals can be set in the context of chronological stimulation through CMV and the resulting T-cell immune response, and is very rarely seen in CMV-seronegatives.

The application of the memory differentiation model on γδ T-cells, rather than αβ T-cells, reveals a pattern that is comparable to the memory phenotype distribution in the CD8+ αβ T-cell compartment. We analyzed additionally all subsets for expression of CD16 as the latter is described as a γδ TCR-independent trigger by opsonized CMV virions and seems to be involved in efficient inhibition of replication [29]. Especially the Vδ2+ compartment displays a heterogeneous pattern of a variety of different memory phenotypes – the composition of the latter differs dominantly when comparing young and old regardless of their CMV serostatus. However, we identified one exception: the rarely described effector memory phenotypes CD27 + CD28-CD45RA + CD16+ and CD27 + CD28-CD45RA + CD16- were affected by both CMV and age. This finding requires further investigation, as usually the Vδ2+ compartment is not described as being involved in anti-CMV-immunity.

The identified signatures in the Vδ2-negative compartment that was subdivided here into Vδ1+ and Vδ1-Vδ2- cells, also revealed influences of both age and CMV on lowering the abundance of early-differentiated phenotypes. We saw marked increases of late-differentiated phenotypes in both groups, associated with both age and CMV-seropositivity, as is the case for CD8+ αβ T-cells [45]. These findings confirm reports generated at “lower resolution”, by only investigating the Vδ2-negative pool [31, 32]. We did not identify the effector phenotype CD27-CD28 + CD45RA ± CD16 ± neither in the Vδ1+ nor in the pool of other (Vδ1-Vδ2-) γδ T-cells, unlike in the Vδ2+ compartment. As for the Vδ1+ compartment, we found a higher median frequency of CD27 + CD28-CD45RA + CD16- cells in CMV-seronegative individuals in the pool of other (Vδ1-Vδ2-) cells and in the Vδ2+ compartment but there only in the young CMV seronegative individuals. Functionality of these cells remains to be determined by further investigations.

Previously reported associations of the Vδ2-negative pool, and early and late differentiated memory phenotypes associated with CMV-specific IgG titer [32], combined with reports describing antibody-dependent anti-CMV activity of γδ T-cells [29], led us to the conclusion that examination of the latter might reveal a functional link between correlations of γδ T-cells and anti-CMV immunity. However, although we found expression of CD16 on minorities of various γδ T-cell subsets, mostly in differentiated phenotypes as earlier reported for the Vδ2 subset [50] we were not able to identify a clear link to CMV-seropositivity.

Memory phenotypes in the CD8+ group of Vδ1+ and Vδ1-Vδ2- γδ T-cells revealed similar associations with age and CMV as found for total γδ T-cells, despite the fact that we identified the presence of a CD28 + CD27- population. Further investigation of CD8+ γδ T-cells is required to investigate whether the expression of the latter is an evolutionary artifact, or of functional importance in anti-CMV immunity as described for αβ CD8+ T-cells [51].

## Conclusions

This study presents a uniquely detailed analysis of the γδ T-cells, in younger and older people with a carefully characterized background. In the same subjects, we also assessed αβ T-cells, and found strong associations of CD8+ αβ T-cells, Vδ1+, other (Vδ1-Vδ2-) with age and also with CMV-seropositivity. CD4:CD8 ratios were lower in old CMV-seropositive than in seronegative individuals. We found increased Vδ1:Vδ2-ratios associated with CMV in the old, similar to what is reported in cancer, supporting the theory of dual reactivity of γδ T-cells. It remains to be determined whether the increased Vδ1+ compartment in CMV-seropositive individuals might have similar detrimental impact as reported for the survival of melanoma patients. The memory differentiation patterns in the Vδ1+ compartment are similar to the CD8+ αβ T-cells markedly changed by age and amplified by the presence of CMV, suggesting an increased memory compartment of acquired immunity over the life-time and in particular in association with CMV. Ongoing work correlating the presented data with multidisciplinary health, social, psychological and genetic data from the BASE-II study will help us better understand the multifactorial immune aging process in a modern society. More functional and longitudinal studies are needed to better understand age-associated immune exhaustion and the role, if any, that a latent CMV infection plays therein due the major investment of immune system resources to maintain control of latent CMV.

## Methods

### Subjects

Subjects participated in the Berlin Aging Study II (BASE-II) with written informed consent and the approval of the Ethics Committee of the Charité-Universitätsmedizin Berlin (approval number EA2/029/09). This study is assembling a uniquely rich database of information on each of the 2200 participating individuals in terms of medical parameters, genetic analyses, socioeconomic, cognitive and psychological status, and will allow us to correlate the immunology reference values reported here with a wide range of factors influencing health status and mortality at follow-up [35]. Cryopreserved peripheral blood mononuclear cells (PBMCs) of 217 participants of this study have been analyzed here. This was a convenience sample randomly selected form the cohort to include younger and older CMV-positive and CMV-negative donors. Here, we included 144 older (62–85 years) and 73 younger (23–35 years) subjects. Fifty-nine of the young and 85 of the old individuals were identified as being CMV-seronegative. Anti-CMV specific IgG titers were determined semi-quantitatively in the subjects’ plasma using the CMV IgG ELISA kit from Omega Diagnostic Group (Scotland).

### Flow cytometry

Cyropreserved PBMC samples were thawed, washed and stained with monoclonal antibodies for the markers of interest, as described in detail in our standardized OMIP-20 panel [34]. Samples were acquired using an LSR II Cytometer (Becton Dickinson). Compensation was automatically performed with single color controls. A biological control was included in each analytical run to ensure comparability between results from different days. Resulting data were analyzed with FlowJo 9.3.2 (Tree Star). The gating strategy is displayed in Additional file 1: Figure S8.

### Statistics

Statistical analysis was performed with Prism 6.d (Graph Pad) and SPSS 22 (IBM). Lymphocyte counts derived from blood count data served as basis for the calculation of the absolute cell counts of the T-cell populations. For this, viable single cells identified in the lymphocyte gate (Additional file 1: Figure S8) were set as equal to the clinically-determined lymphocyte counts. The Mann-Whitney U test was used to compare cell frequencies between the different groups of interest. P-values were corrected for multiple testing using the Bonferroni method.

## Additional file

Additional file 1: Table S1. Results of Mann‐Whitney comparisons of the γδ T‐cell compartments and subsets. **Table S2.** Results of Mann‐Whitney comparisons of the CD8+ γδ T‐cell compartments and subsets. **Table S3.** Results of Mann‐Whitney comparisons of the αβT‐cell compartments and subsets. **Table S4.** Results of Mann‐Whitney comparisons of the absolute counts for all T‐cell compartments. **Figure S1.** Median frequencies and absolute counts of the γδ T‐cell compartments in young and old CMV‐seropositive (CMV+) and seronegative (CMV‐) individuals. **Figure S2.** Differentiation phenotypes of the Vδ2+ compartment. **Figure S3.** Differentiation phenotypes of the Vδ1+ compartment. **Figure S4.** Differentiation phenotypes of the Vδ1‐Vδ2‐compartment. **Figure S5.** Differentiation phenotypes of the CD8+ γδ T‐cells. **Figure S6.** Differentiation phenotypes of the CD8+ αβT‐cells. **Figure S7.** Differentiation phenotypes of the CD4 + αβT‐cells. **Figure S8.** Gating Strategy T‐cells. (PDF 1365 kb)

## Abbreviations

BASE: Berlin Aging Study
CMV: cytomegalovirus
EPCR: endothelial protein C receptor
MHC: major histocompatibility complex
MICA/B: MHC class I polypeptide related sequence A/B
OMIP: orchestrating multiplexity in polychromatic sciences
PBMC: peripheral blood mononuclear cell
TCR: T-cell receptor
TEMRA: effector memory T-cells re-expressing CD45RA
