# Supplementary material for: Peripheral blood T-cell signatures from high-resolution immune phenotyping of γδ and αβ T-cells in younger and older subjects in the Berlin Aging Study II
Source: Immun Ageing. 2015 Dec 4;12:25. doi: 10.1186/s12979-015-0052-x (PMC4670504; doi:10.1186/s12979-015-0052-x)

Peripheral blood T-cell reference values from high-resolution immune phenotyping of γδ and αβ T-cells in younger and older subjects in the Berlin Aging Study II

Kilian Wistuba-Hamprecht, Karin Haehnel, Nicole Janssen, Ilja Demuth, Graham Pawelec

Supplementary Material

Table S1: Results of Mann-Whitney comparisons of the γδ T-cell compartments and subsets.

p-values and median frequencies are displayed for young CMV-seropositive (yCMV+), young CMV-seronegative (yCMV-), old CMV-seropositive (oCMV+) and old CMV-seronegative (oCMV-).

phenotype		yCMV- vs oCMV-		yCMV+ vs oCMV+		yCMV- vs yCMV+		oCMV- vs oCMV+		yCMV- vs oCMV+		oCMV- vs yCMV+	
**γδ T-cells/CD3+**	p -value	**< 0.0001**		0.4865		0.2179		0.353		**0.0057**		0.156	
	median frequencies	3.84	2.13	2.58	2.31	3.84	2.58	2.13	2.31	3.84	2.31	2.13	2.58
**Vδ2+/γδ T-cells**	p -value	0.1438		0.0691		0.2976		**< 0.0001**		**< 0.0001**		0.726	
	median frequencies	71.49	66.30	68.61	37.88	71.49	68.61	66.30	37.88	71.49	37.88	66.3	68.61
**CD27+CD28+CD45RA+CD16+/Vδ2+**		n.a.											
**CD27+CD28+CD45RA+CD16-/Vδ2+**	p -value	0.9871		0.2988		0.2801		0.824		0.8249		0.2921	
	median frequencies	12.83	12.43	8.43	11.96	12.83	8.43	12.43	11.96	12.83	11.96	12.43	8.43
**CD27+CD28+CD45RA-CD16+/Vδ2+**		n.a.											
**CD27+CD28+CD45RA-CD16-/Vδ2+**	p -value	0.0493		0.1733		0.5912		0.2567		**0.0004**		0.5261	
	median frequencies	22.71	30.07	25.76	36.10	22.71	25.76	30.07	36.10	22.71	36.10	30.07	25.76
**CD27+CD28-CD45RA+CD16+/Vδ2+**	p -value	0.0207		0.5185		**0.0054**		0.204		**0.0015**		0.087	
	median frequencies	0.11	0.05	0.01	0.04	0.11	0.01	0.05	0.04	0.11	0.04	0.05	0.01
**CD27+CD28-CD45RA+CD16-/Vδ2+**	p -value	**< 0.0001**		0.0903		**0.0022**		0.0603		**< 0.0001**		0.7623	
	median frequencies	28.17	17.46	17.49	12.59	28.17	17.49	17.46	12.59	28.17	12.59	17.46	17.49
**CD27+CD28-CD45RA-CD16+/Vδ2+**		n.a.											
**CD27+CD28-CD45RA-CD16-/Vδ2+**	p -value	0.2856		0.1739		0.0742		0.0194		0.2977		0.0243	
	median frequencies	5.43	4.20	7.88	6.03	5.43	7.88	4.20	6.03	5.43	6.03	4.20	7.88
**CD27-CD28+CD45RA+CD16+/Vδ2+**		n.a.											
**CD27-CD28+CD45RA+CD16-/Vδ2+**	p -value	0.1272		0.369		0.2187		0.3279		0.8108		0.9489	
	median frequencies	1.27	1.56	1.55	1.18	1.27	1.55	1.56	1.18	1.27	1.18	1.56	1.55
**CD27-CD28+CD45RA-CD16+/Vδ2+**		n.a.											
**CD27-CD28+CD45RA-CD16-/Vδ2+**	p -value	**0.0005**		0.4009		0.3303		0.9271		0.0087		0.446	
	median frequencies	2.76	4.64	4.48	5.49	2.76	4.48	4.64	5.49	2.76	5.49	4.64	4.48
**CD27-CD28-CD45RA+CD16+/Vδ2+**	p -value	0.6109		0.3201		0.4664		0.4664		0.7125		0.3198	
	median frequencies	0.14	0.18	0.08	0.15	0.14	0.08	0.18	0.15	0.14	0.15	0.18	0.08
**CD27-CD28-CD45RA+CD16-/Vδ2+**	p -value	0.9834		0.7768		0.7749		0.6014		0.327		0.8777	
	median frequencies	15.60	14.95	11.47	10.76	15.60	11.47	14.95	10.76	15.60	10.76	14.95	11.47
**CD27-CD28-CD45RA-CD16+/Vδ2+**		n.a.											
**CD27-CD28-CD45RA-CD16-/Vδ2+**	p -value	0.7188		0.6153		0.0392		0.0392		0.0447		0.0601	
	median frequencies	2.62	2.68	3.71	4.27	2.62	3.71	2.68	4.27	2.62	4.27	2.68	3.71
**Vδ1+/γδ T-cells**	p -value	0.8003		0.1125		0.1487		**< 0.0001**		**< 0.0001**		0.1822	
	median frequencies	12.32	11.46	11.31	32.93	12.32	32.93	11.46	32.93	12.32	32.93	11.46	11.31
**CD27+CD28+CD45RA+CD16+/Vδ1+**	p -value	0.1619		0.5522		0.4549		0.2453		0.8474		0.8696	
	median frequencies	0.03	0.05	0.07	0.02	0.03	0.07	0.05	0.02	0.03	0.02	0.05	0.07
**CD27+CD28+CD45RA+CD16-/Vδ1+**	p -value	**< 0.0001**		0.0391		0.0122		**0.0045**		**< 0.0001**		0.842	
	median frequencies	29.14	17.70	16.35	6.33	29.14	16.35	17.70	6.33	29.14	6.33	17.70	16.35
**CD27+CD28+CD45RA-CD16+/Vδ1+**		n.a.											
**CD27+CD28+CD45RA-CD16-/Vδ1+**	p -value	0.9352		0.6664		0.0797		0.0102		**0.0015**		0.1424	
	median frequencies	5.11	5.89	4.22	2.15	5.11	4.22	5.89	2.15	5.11	2.15	5.89	4.22
**CD27+CD28-CD45RA+CD16+/Vδ1+**	p -value	0.3458		0.5323		0.4977		0.4287		0.7999		0.9314	
	median frequencies	0.06	0.03	0.04	0.05	0.06	0.04	0.03	0.05	0.06	0.05	0.03	0.04
**CD27+CD28-CD45RA+CD16-/Vδ1+**	p -value	0.1011		0.0288		**0.0048**		**< 0.0001**		**< 0.0001**		0.056	
	median frequencies	37.81	32.81	24.26	17.77	37.81	17.77	32.81	17.77	37.81	17.77	32.81	17.77
**CD27+CD28-CD45RA-CD16+/Vδ1+**		n.a.											
**CD27+CD28-CD45RA-CD16-/Vδ1+**	p -value	0.056		0.0173		0.7954		**0.0022**		**< 0.0001**		0.4411	
	median frequencies	1.80	1.31	1.56	0.69	1.80	1.56	1.31	0.69	1.80	0.69	1.31	1.56
**CD27-CD28+CD45RA+CD16+/Vδ1+**		n.a.											
**CD27-CD28+CD45RA+CD16-/Vδ1+**		n.a.											
**CD27-CD28+CD45RA-CD16+/Vδ1+**		n.a.											
**CD27-CD28+CD45RA-CD16-/Vδ1+**		n.a.											
**CD27-CD28-CD45RA+CD16+/Vδ1+**	p -value	0.0159		0.6875		0.2981		0.871		0.0174		0.7255	
	median frequencies	0.34	0.65	0.46	0.74	0.34	0.46	0.65	0.74	0.34	0.74	0.65	0.46
**CD27-CD28-CD45RA+CD16-/Vδ1+**	p -value	**< 0.0001**		0.0275		**0.0018**		**< 0.0001**		**< 0.0001**		0.3885	
	median frequencies	18.65	37.02	46.87	65.15	18.65	46.87	37.02	65.15	18.65	65.15	37.01	46.87
**CD27-CD28-CD45RA-CD16+/Vδ1+**	p -value	0.4103		0.6057		0.6705		0.6242		0.2102		0.8439	
	median frequencies	0.06	0.02	0.03	0.01	0.06	0.03	0.02	0.01	0.06	0.01	0.01	0.03
**CD27-CD28-CD45RA-CD16-/Vδ1+**	p -value	0.8439		0.992		0.5574		0.5574		0.6291		0.5604	
	median frequencies	0.93	1.38	1.44	0.97	0.93	1.44	1.38	0.97	0.93	0.97	1.38	1.44
**Vδ1-Vδ2-/γδ T-cells**	p -value	0.1563		0.555		0.3605		0.3028		0.063		0.9269	
	median frequencies	14.94	18.35	17.17	18.91	14.94	17.17	18.35	18.91	14.94	18.91	18.35	17.17
**CD27+CD28+CD45RA+CD16+/Vδ1-Vδ2-**	p -value	0.2828		0.0145		0.1457		0.3771		0.0359		0.0855	
	median frequencies	0.04	0.0	0.14	0.0	0.04	0.14	0.0	0.0	0.04	0.0	0.0	0.14
**CD27+CD28+CD45RA+CD16-/Vδ1-Vδ2-**	p -value	**< 0.0001**		0.0462		0.2247		0.1554		**< 0.0001**		0.1136	
	median frequencies	29.26	18.57	23.75	14.31	29.26	23.75	18.57	14.31	29.26	14.31	18.57	23.75
**CD27+CD28+CD45RA-CD16+/Vδ1-Vδ2-**		n.a.											
**CD27+CD28+CD45RA-CD16-/Vδ1-Vδ2-**	p -value	0.6112		0.457		0.4246		0.0286		**0.0071**		0.6269	
	median frequencies	6.10	5.14	5.42	4.10	6.10	5.42	5.14	4.10	6.10	4.10	5.14	5.42
**CD27+CD28-CD45RA+CD16+/Vδ1-Vδ2-**	p -value	0.1661		> 0.9999		0.5596		0.0206		0.1876		0.0979	
	median frequencies	0.06	0.10	0.07	0.06	0.06	0.07	0.10	0.06	0.06	0.06	0.10	0.07
**CD27+CD28-CD45RA+CD16-/Vδ1-Vδ2-**	p -value	**0.0057**		0.9272		0.0138		0.2279		**0.0005**		0.4824	
	median frequencies	24.63	19.59	19.00	16.26	24.63	19.00	19.59	16.26	24.63	16.26	19.59	19.00
**CD27+CD28-CD45RA-CD16+/Vδ1-Vδ2-**		n.a.											
**CD27+CD28-CD45RA-CD16-/Vδ1-Vδ2-**	p -value	0.0346		**0.0064**		0.8951		0.0836		**< 0.0001**		0.3055	
	median frequencies	1.42	0.75	1.50	0.55	1.42	1.50	0.75	0.55	1.42	0.55	0.75	1.50
**CD27-CD28+CD45RA+CD16+/Vδ1-Vδ2-**		n.a.											
**CD27-CD28+CD45RA+CD16-/Vδ1-Vδ2-**		n.a.											
**CD27-CD28+CD45RA-CD16+/Vδ1-Vδ2-**		n.a.											
**CD27-CD28+CD45RA-CD16-/Vδ1-Vδ2-**		n.a.											
**CD27-CD28-CD45RA+CD16+/Vδ1-Vδ2-**	p -value	**0.0006**		0.1434		0.3897		0.8437		**0.0009**		0.2041	
	median frequencies	0.50	1.15	0.81	1.24	0.50	0.81	1.15	1.24	0.50	1.24	1.15	0.81
**CD27-CD28-CD45RA+CD16-/Vδ1-Vδ2-**	p -value	**< 0.0001**		0.0592		0.0144		0.0178		**< 0.0001**		0.5097	
	median frequencies	25.57	39.93	37.14	48.13	25.57	37.14	39.93	48.13	25.57	48.13	39.93	37.14
**CD27-CD28-CD45RA-CD16+/Vδ1-Vδ2-**	p -value	0.5173		0.8257		0.943		0.3585		0.7384		0.7417	
	median frequencies	0.04	0.04	0.05	0.0	0.04	0.05	0.04	0.0	0.04	0.0	0.04	0.05
**CD27-CD28-CD45RA-CD16-/Vδ1-Vδ2-**	p -value	**0.0002**		0.6561		0.3494		0.8755		**0.0006**		0.489	
	median frequencies	2.35	4.34	3.88	4.28	2.35	3.88	4.34	4.28	2.35	4.28	4.34	3.88
**Vδ1+/Vδ2+**	p -value	0.5722		0.0634		0.1754		**< 0.0001**		**< 0.0001**		0.334	
	median	0.19	0.17	0.20	0.83	0.19	0.20	**0.17**	**0.83**	**0.19**	**0.83**	**0.17**	0.20

Bonferroni correction: p-values ≤0.0083 were considered significant

Table S2: Results of Mann-Whitney comparisons of the CD8+ γδ T-cell compartments and subsets.

p-values and median frequencies are displayed for young CMV-seropositive (yCMV+), young CMV-seronegative (yCMV-), old CMV-seropositive (oCMV+) and old CMV-seronegative (oCMV-).

phenotype		oCMV+ vs oCMV-	yCMV+ vs yCMV-	oCMV+ vs yCMV+	oCMV- vs yCMV-	oCMV+ vs yCMV-	oCMV- vs yCMV+
CD8+/γδ T-cells	p -value	**0.0008**	0.3605	0.1672	0.5323	**0.0002**	0.6131
	median frequencies	10 5.2	7.1 4.9	10 7.1	5.2 4.9	10 4.9	5.2 7.1
Vδ2+/γδ T-cells	p -value	**0.0026**	0.0676	0.9293	0.2563	0.01	0.0295
	median frequencies	1.5 6.9	2.5 4.6	1.5 2.5	6.9 4.6	1.5 4.6	6.9 2.5
CD27-CD28-/Vδ2+		n.a.					
CD27-CD28+/Vδ2+		n.a.					
CD27+CD28-/Vδ2+		n.a.					
CD27+CD28+/Vδ2+		n.a.					
Vδ1+/γδ T-cells	p -value	**0.0062**	0.2062	0.1221	0.0245	**< 0.0001**	0.9023
	median frequencies	65.5 55.6	54.6 47.5	65.5 54.6	55.6 47.5	65.5 47.5	55.6 54.6
CD27-CD28-/Vδ1+	p -value	**0.0002**	**0.0029**	0.2143	0.0217	**< 0.0001**	0.2519
	median frequencies	73.0 60.0	63.5 47.9	73.0 63.5	60.0 47.9	73.0 47.9	60.0 63.5
CD27-CD28+/Vδ1+	p -value	0.6671	0.7437	0.8928	0.8323	0.7417	0.5812
	median frequencies	1.5 1.6	1.6 1.6	1.5 1.6	1.6 1.6	1.5 1.6	1.6 1.6
CD27+CD28-/Vδ1+	p -value	0.0162	0.0324	0.327	**0.0058**	**< 0.0001**	0.5319
	median frequencies	15.4 24.8	17.8 32.6	15.4 17.8	24.8 32.6	15.4 32.6	24.8 17.8
CD27+CD28+/Vδ1+	p -value	**0.0002**	0.014	0.2136	**0.001**	**< 0.0001**	0.4634
	median frequencies	3.0 5.7	5.3 9.3	3.0 5.3	5.7 9.3	3.0 9.3	5.7 5.3
Vδ1-Vδ2-/γδ T-cells	p -value	0.7348	0.5247	0.0453	**0.0002**	**0.0005**	0.0358
	median frequencies	22.4 23.5	32.6 36.0	22.4 32.6	23.5 36.0	22.4 36.0	23.5 32.6
CD27-CD28-/Vδ1-Vδ2-	p -value	0.0357	**0.006**	0.0328	**< 0.0001**	**< 0.0001**	0.7036
	median frequencies	53.2 29.3	17.5 7.5	53.2 17.5	29.3 7.5	53.2 7.5	29.3 17.5
CD27-CD28+/Vδ1-Vδ2-	p -value	0.2367	0.0567	0.5949	**< 0.0001**	**0.0027**	0.1376
	median frequencies	1.3 1.6	1.0 0.7	1.3 1.0	1.6 1.0	1.3 0.7	1.6 1.0
CD27+CD28-/Vδ1-Vδ2-	p -value	0.0513	0.6587	0.4447	0.1732	0.2582	0.6149
	median frequencies	8.5 12.6	13.7 9.7	8.5 13.7	12.6 9.7	8.5 9.7	12.6 13.7
CD27+CD28+/Vδ1-Vδ2-	p -value	0.1561	0.0744	**0.001**	**< 0.0001**	**< 0.0001**	0.0222
	median frequencies	21.6 37.6	57.4 70.0	21.6 57.4	37.6 70.0	21.6 70.0	37.6 57.4

Bonferroni correction: p-values ≤0.0083 were considered significant

Table S3: Results of Mann-Whitney comparisons of the αβT-cell compartments and subsets.

αβ T-cells are identified as CD3+ γδ TCR-. p-values and median frequencies are displayed for young CMV-seropositive (yCMV+), young CMV-seronegative (yCMV-), old CMV-seropositive (oCMV+) and old CMV-seronegative (oCMV-).

phenotype		yCMV- vs oCMV-		yCMV+ vs oCMV+		yCMV- vs yCMV+		oCMV- vs oCMV+		yCMV- vs oCMV+		oCMV- vs yCMV+	
γδ TCR-/CD3+	p -value	**< 0.0001**		0.5357		0.1989		0.2706		**0.0076**		0.1883	
	median frequencies	95.6	97.4	96.9	97.1	95.6	96.9	97.4	97.1	95.6	97.1	97.4	96.9
CD4+/γδ TCR-	p -value	**< 0.0001**		0.0260		0.4939		**< 0.0001**		**0.0002**		**< 0.0001**	
	median frequencies	69.8	81.1	66.2	74.9	69.8	66.2	81.1	74.9	69.8	74.9	81.1	66.2
CD27+CD28+CD45RA+CD16-/CD4+	p -value	**< 0.0001**		0.0120		0.0363		**0.0034**		**< 0.0001**		0.2520	
	median frequencies	68.7	57.9	61.6	52.1	68.7	61.6	57.9	52.1	68.7	52.1	57.9	61.6
CD27+CD28+CD45RA-CD16-/CD4+	p -value	**< 0.0001**		0.0243		0.2630		0.484		**< 0.0001**		0.0521	
	median frequencies	28.2	38.2	32.2	39.2	28.2	32.2	38.2	39.2	28.2	39.2	38.2	32.2
CD27+CD28-CD45RA+CD16-/CD4+	p -value	0.1934		0.3510		0.0201		**< 0.0001**		**< 0.0001**		0.1460	
	median frequencies	0.3	0.4	0.6	1.0	0.3	0.6	0.4	1.0	0.3	1.0	0.4	0.6
CD27+CD28-CD45RA-CD16-/CD4+	p -value	**0.0001**		0.0173		0.0292		**< 0.0001**		**< 0.0001**		0.8338	
	median frequencies	0.07	0.13	0.13	0.25	0.07	0.13	0.13	0.25	0.07	0.25	0.13	0.013
CD27-CD28+CD45RA+CD16-/CD4+	p -value	0.5235		0.9740		0.5544		0.1150		0.3923		0.2847	
	median frequencies	0.10	0.10	0.13	0.12	0.10	0.13	0.10	0.13	0.10	0.12	0.10	0.13
CD27-CD28+CD45RA-CD16-/CD4+	p -value	0.0314		0.8988		0.0120		0.0143		**0.0001**		0.0956	
	median frequencies	2.57	2.83	3.7	3.98	2.57	3.7	2.83	3.98	2.57	3.98	2.83	3.7
CD27-CD28-CD45RA-CD16-/CD4+	p -value	**0.0029**		**0.0053**		**0.0002**		**< 0.0001**		**< 0.0001**		0.0138	
	median frequencies	0.04	0.06	0.22	1.12	0.04	0.22	0.06	1.12	0.04	1.12	0.06	2.22
CD27-CD28-CD45RA+CD16-/CD4+	p -value	0.0150		0.0290		0.0295		**< 0.0001**		**< 0.0001**		0.1425	
	median frequencies	0.02	0.05	0.11	0.56	0.02	0.11	0.05	0.56	0.02	0.56	0.05	0.11
CD8+/γδ TCR-	p -value	**< 0.0001**		0.0583		0.7376		**< 0.0001**		**0.0003**		**< 0.0001**	
	median frequencies	27.10	16.23	30.42	21.54	27.10	30.42	16.23	21.54	27.10	21.54	16.23	30.42
CD27+CD28+CD45RA+CD16-/CD8+	p -value	**< 0.0001**		**0.0001**		**0.0029**		**0.0005**		**< 0.0001**		0.0115	
	median frequencies	76.54	46.10	65.20	33.93	76.54	65.20	46.10	33.93	76.54	33.93	46.10	65.20
CD27+CD28+CD45RA-CD16-/CD8+	p -value	**< 0.0001**		**0.0057**		0.8875		**0.0009**		**< 0.0001**		**< 0.0001**	
	median frequencies	11.23	24.39	12.27	16.66	11.23	12,2700	24.39	16.66	11.23	16.66	24.39	12.27
CD27+CD28-CD45RA+CD16-/CD8+	p -value	**0.0031**		0.3548		0.1087		0.0121		0.7876		0.4089	
	median frequencies	4.19	6.44	5.32	4.27	4.19	5.32	6.44	4.27	4.19	4.27	6.44	5.32
CD27+CD28-CD45RA-CD16-/CD8+	p -value	0.0352		0.4358		0.2286		0.0334		0.8229		0.7555	
	median frequencies	1.21	1.69	1.99	1.17	1.21	1.99	1.69	1.17	1.21	1.17	1.69	1.99
CD27-CD28+CD45RA+CD16-/CD8+	p -value	**< 0.0001**		**0.0035**		0.1996		0.0243		**< 0.0001**		0.0599	
	median frequencies	0.45	1.07	0.80	1.43	0.45	0.80	1.07	1.43	0.45	1.43	1.07	0.80
CD27-CD28+CD45RA-CD16-/CD8+	p -value	**< 0.0001**		**0.0033**		0.3786		0.1564		**< 0.0001**		**0.0002**	
	median frequencies	0.73	1.93	0.81	1.83	0.73	0.81	1.93	1.83	0.73	1.83	1.93	0.81
CD27-CD28-CD45RA+CD16-/CD8+	p -value	**< 0.0001**		0.0113		**< 0.0001**		**< 0.0001**		**< 0.0001**		0.0140	
	median frequencies	2.52	6.85	12.55	25.32	2.52	12.55	6.85	25.32	2.52	25.32	6.85	12.55
CD27-CD28-CD45RA-CD16-/CD8+	p -value	0.0204		0.7475		**0.0018**		**0.0001**		**< 0.0001**		0.0415	
	median frequencies	0.55	0.96	3.01	2.05	0.55	3.01	0.96	2.05	0.55	2.05	0.96	3.01
CD4/CD8	p -value	**< 0.0001**		0.0497		0.6148		**< 0.0001**		**0.0003**		**< 0.0001**	
	median	2.59	5.01	2.21	3.43	2.59	2.21	5.01	3.43	2.59	3.43	5.01	2.21

Bonferroni correction: p-values ≤0.0083 were considered significant

Table S4: Results of Mann-Whitney comparisons of the absolute counts for all T-cell compartments.

p-values and median counts are displayed for young CMV-seropositive (yCMV+), young CMV-seronegative (yCMV-), old CMV-seropositive (oCMV+) and old CMV-seronegative (oCMV-).

phenotype		yCMV- vs oCMV-		yCMV+ vs oCMV+		yCMV- vs yCMV+		oCMV- vs oCMV+		yCMV- vs oCMV+		oCMV- vs yCMV+	
**Lymphocytes**	p -value	**0.0002**		0.9798		0.1230		0.1504		0.1045		0.3460	
	median counts	1815	1540	1700	1670	1815	1700	1540	1670	1815	1670	1540	1700
**T-cells**	p -value	**< 0.0001**		0.8504		0.1255		0.0585		0.0488		0.1247	
	median counts	1449	1119	1354	1323	1449	1354	1119	1323	1449	1323	1119	1354
**αβ T-cells**	p -value	**< 0.0001**		0.8504		0.1808		0.0708		0.0856		0.1617	
	median counts	1400	1099	1295	1276	1400	1295	1099	1276	1400	1276	1099	1295
**CD4+ T-cells**	p -value	0.0708		0.4497		0.1803		0.6640		0.6227		0.6701	
	median counts	960	900	848	914	960	848	900	914	960	914	900	848
**CD8+ T-cells**	p -value	**< 0.0001**		0.0758		0.5311		**< 0.0001**		**0.0003**		**< 0.0001**	
	median counts	378	171	246	299	378	246	171	299	378	299	171	246
**CD4/CD8**	p -value	**< 0.0001**		0.0497		0.6859		**< 0.0001**		**0.0002**		**< 0.0001**	
	median	2.58	5.07	2.21	3.43	2.58	2.21	5.07	3.43	2.58	3.43	5.07	2.21
**γδ T-cells/CD3+**	p -value	**< 0.0001**		0.4063		0.1034		0.1185		**0.0006**		0.0687	
	median counts	51	25	40	30	51	40	25	30	51	30	25	40
**Vδ2+ T-cells**	p -value	**< 0.0001**		0.0742		0.0267		0.0623		**< 0.0001**		0.4821	
	median counts	37	14	24	10	37	24	14	10	37	10	14	24
**Vδ1+ T-cells**	p -value	**< 0.0001**		0.6345		0.8939		**< 0.0001**		0.1349		**0.0049**	
	median counts	**6**	**2**	5	8	6	5	**2**	**8**	6	8	2	**5**
**Vδ1-Vδ2- T-cells**	p -value	**< 0.0001**		0.5672		0.2588		**0.0051**		0.0181		0.0197	
	median counts	**7**	**4**	6	5	7	6	**4**	**5**	7	5	4	6
**Vδ1+/Vδ2+**	p -value	0.6293		0.0641		0.1496		**< 0.0001**		**< 0.0001**		0.2794	
	median	0.18	0.17	0.20	0.83	0.18	0.20	0.17	0.83	0.18	0.83	0.17	0.20

Bonferroni correction: p-values ≤0.0083 were considered significant

Figure S1

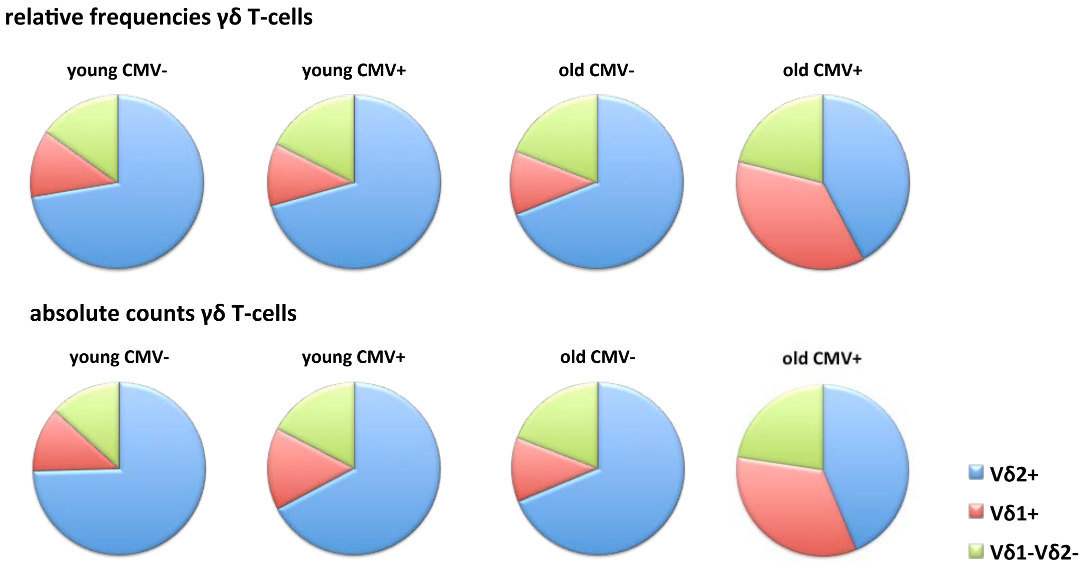

Figure S1: Median frequencies and absolute counts of the γδ T-cell compartments in young and old CMV-seropositive (CMV+) and seronegative (CMV-) individuals.

Figure S2

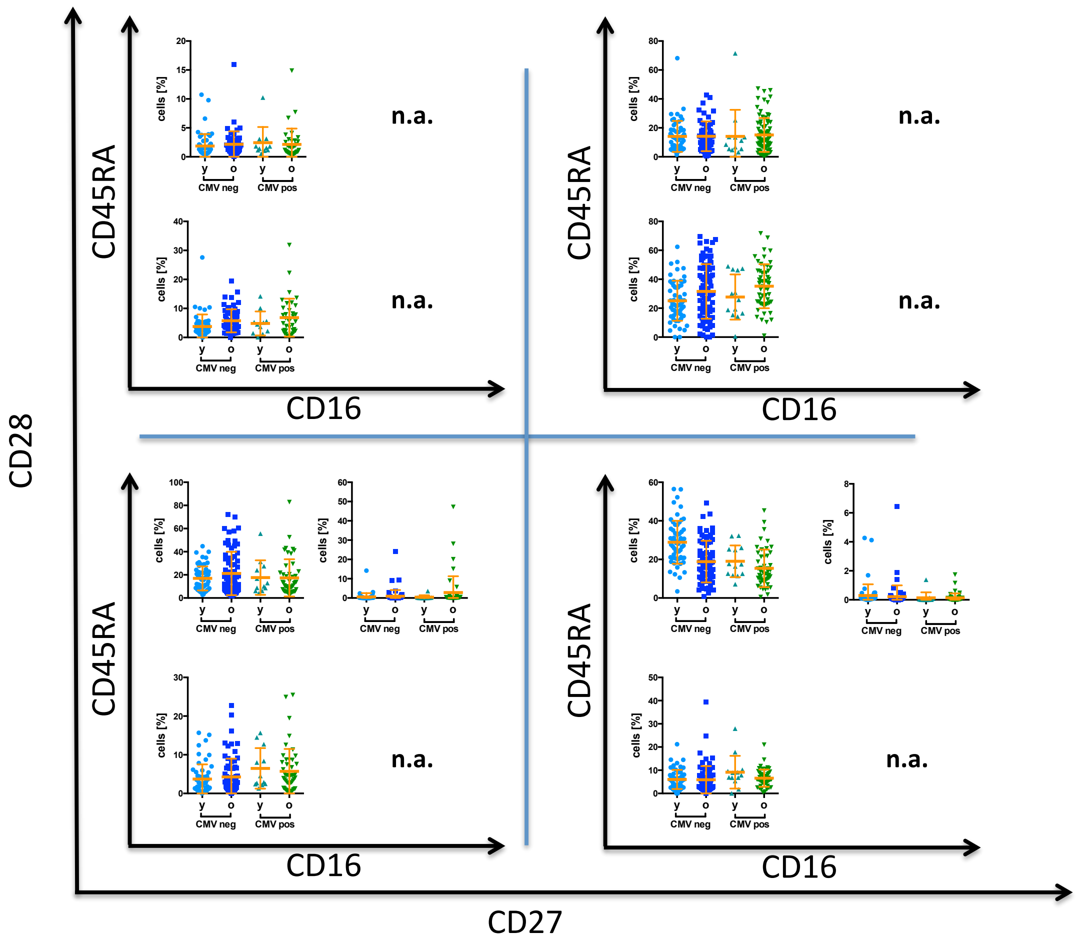

Figure S2: Differentiation phenotypes of the Vδ2+ compartment

The differentiation statuses were identified by CD27, CD28, CD45RA and CD16 in young (y) and old (o) CMV seropositive (CMV pos) and seronegative (CMV neg) individuals.

Figure S3

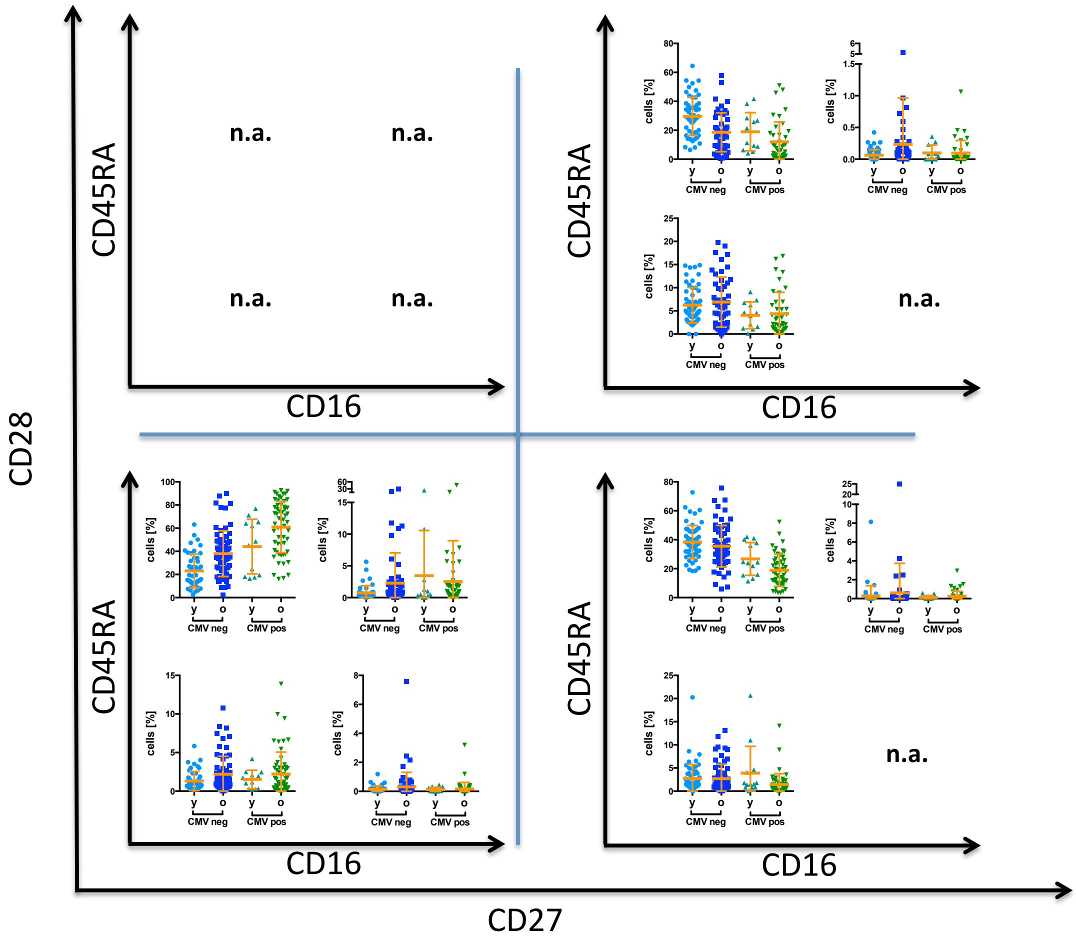

Figure S3: Differentiation phenotypes of the Vδ1+ compartment

The differentiation statuses were identified by CD27, CD28, CD45RA and CD16 in young (y) and old (o) CMV seropositive (CMV pos) and seronegative (CMV neg) individuals.

Figure S4

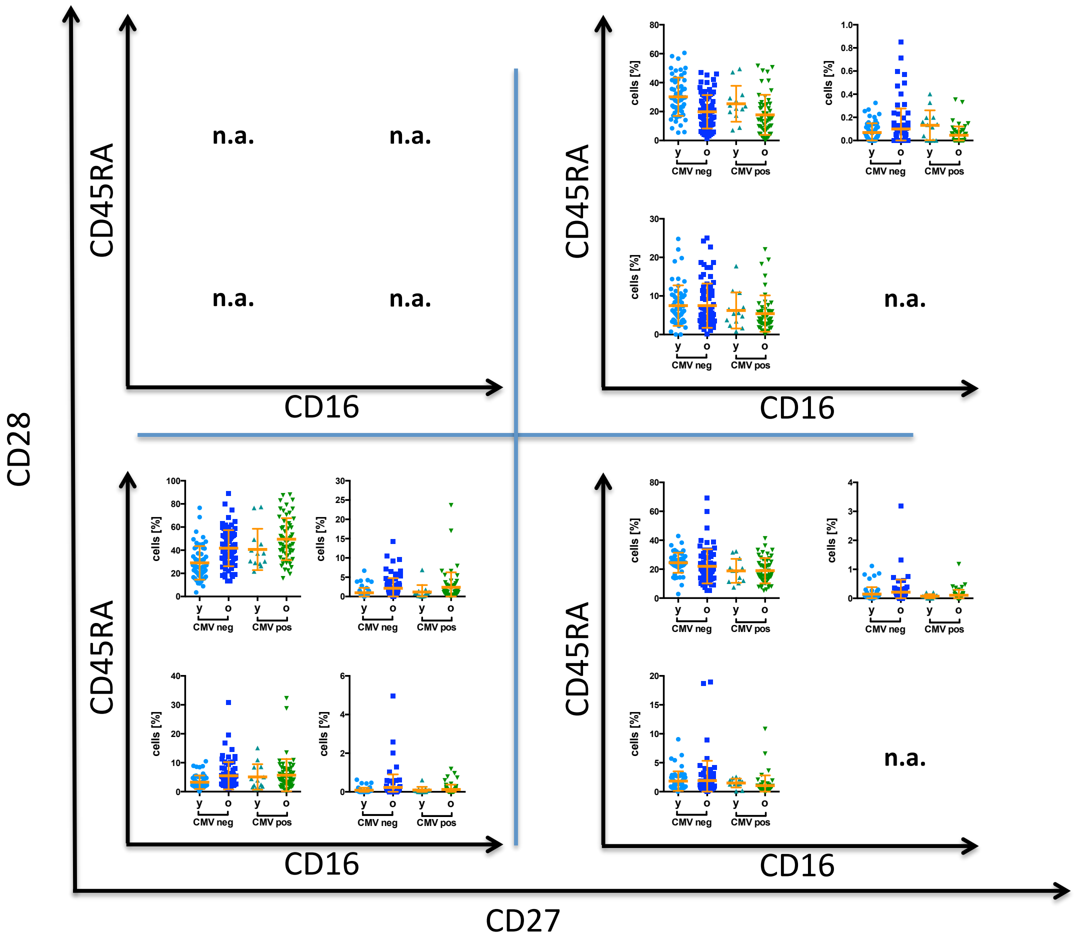

Figure S4: Differentiation phenotypes of the Vδ1-Vδ2- compartment

The differentiation statuses were identified by CD27, CD28, CD45RA and CD16 in young (y) and old (o) CMV seropositive (CMV pos) and seronegative (CMV neg) individuals.

Figure S5

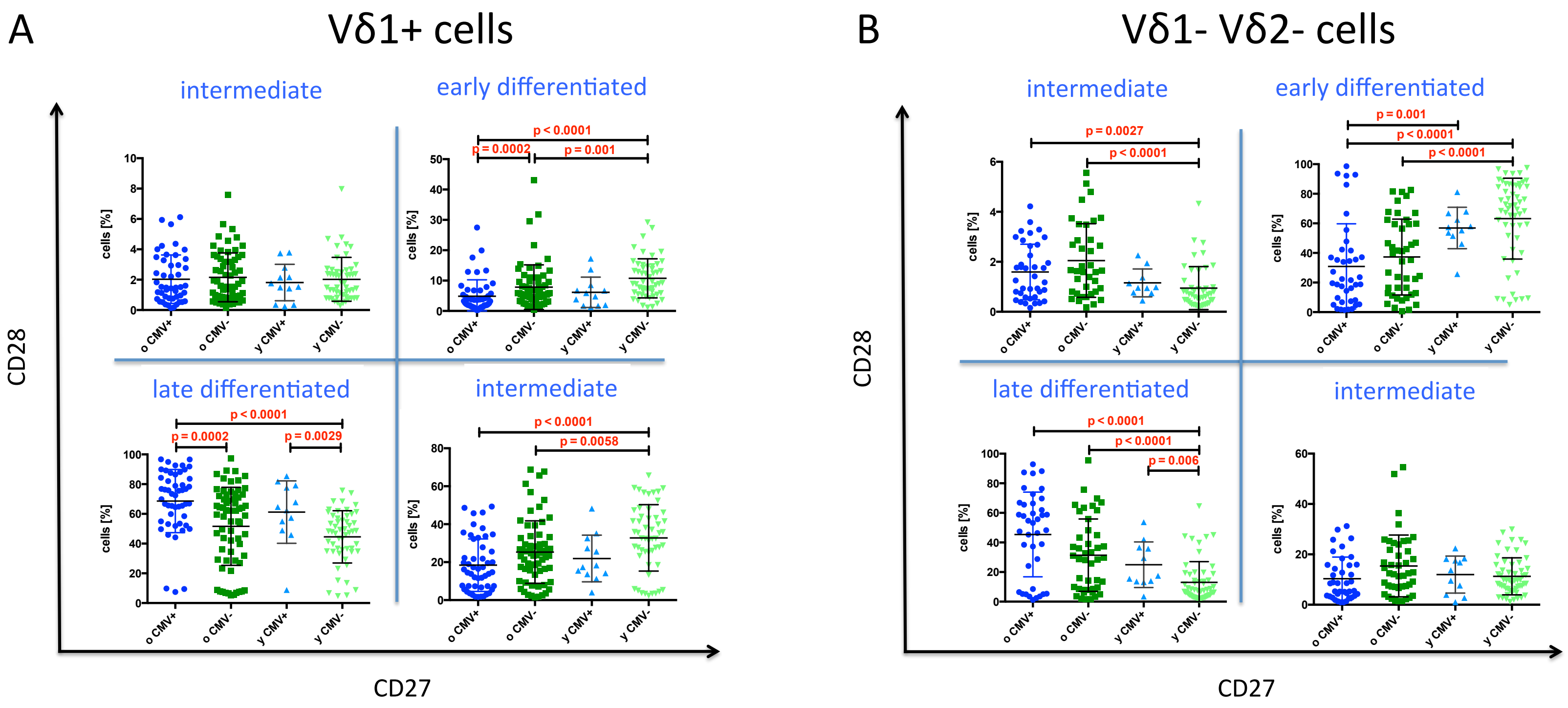

Figure S5: Differentiation phenotypes of the CD8+ γδ T-cells

The differentiation statuses of the **A** Vδ1+ and **B** Vδ1-Vδ2- compartment were identified by CD27 and CD28 in young (y) and old (o) CMV seropositive (CMV+) and seronegative (CMV-) individuals.

Mann Whitney U test was used for the statistical comparison of groups. Bonferroni correction adjusted p≤0.0083 as the level of significance.

Figure S6

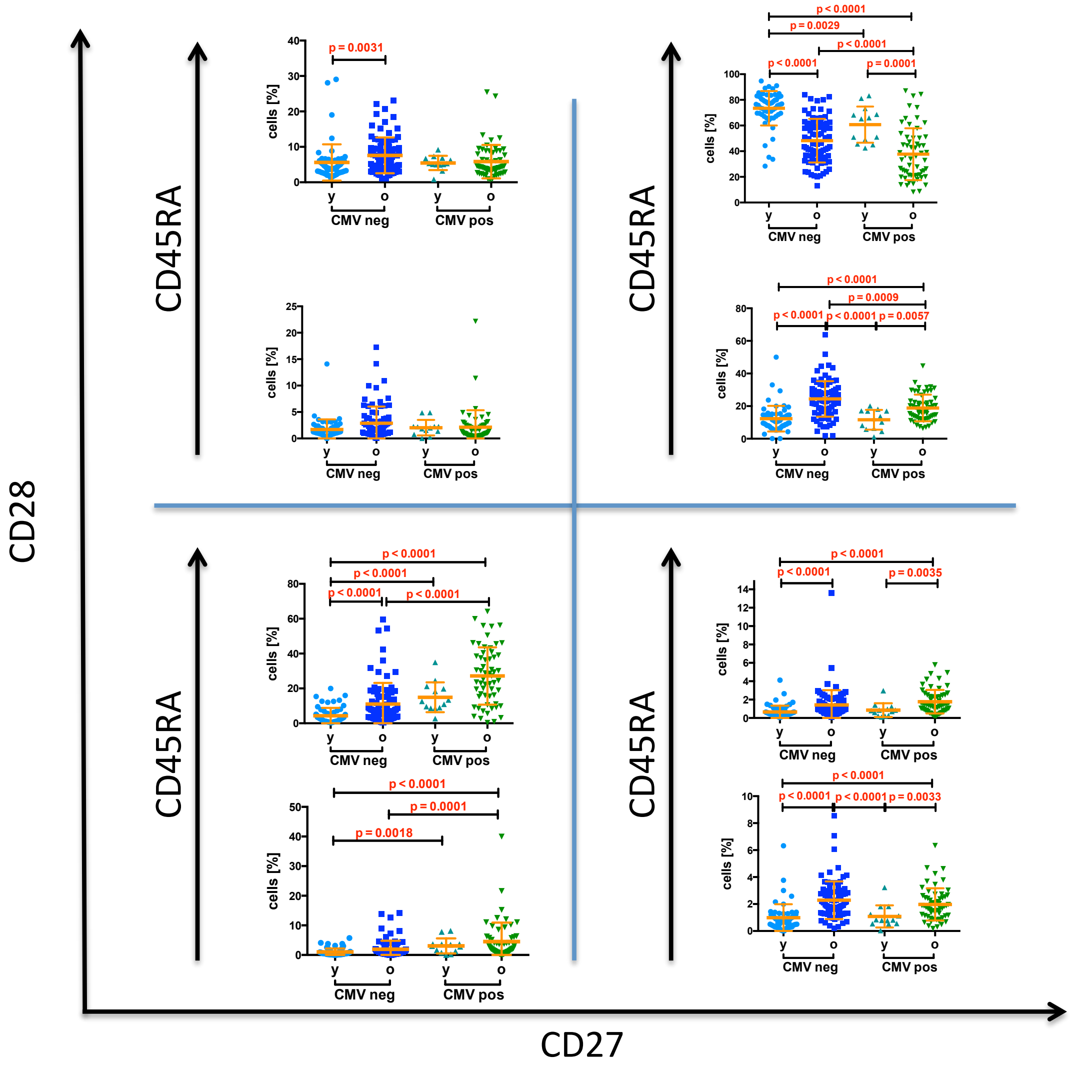

Figure S6: Differentiation phenotypes of the CD8+ αβ T-cells

The differentiation compartments were identified by CD27, CD28, CD45RA and were all CD16 negative in young (y) and old (o) CMV seropositive (CMV pos) and seronegative (CMV neg) individuals.

Mann Whitney U test was used for the statistical comparison of groups.

Bonferroni correction adjusted p≤0.0083 as the level of significance.

Figure S7

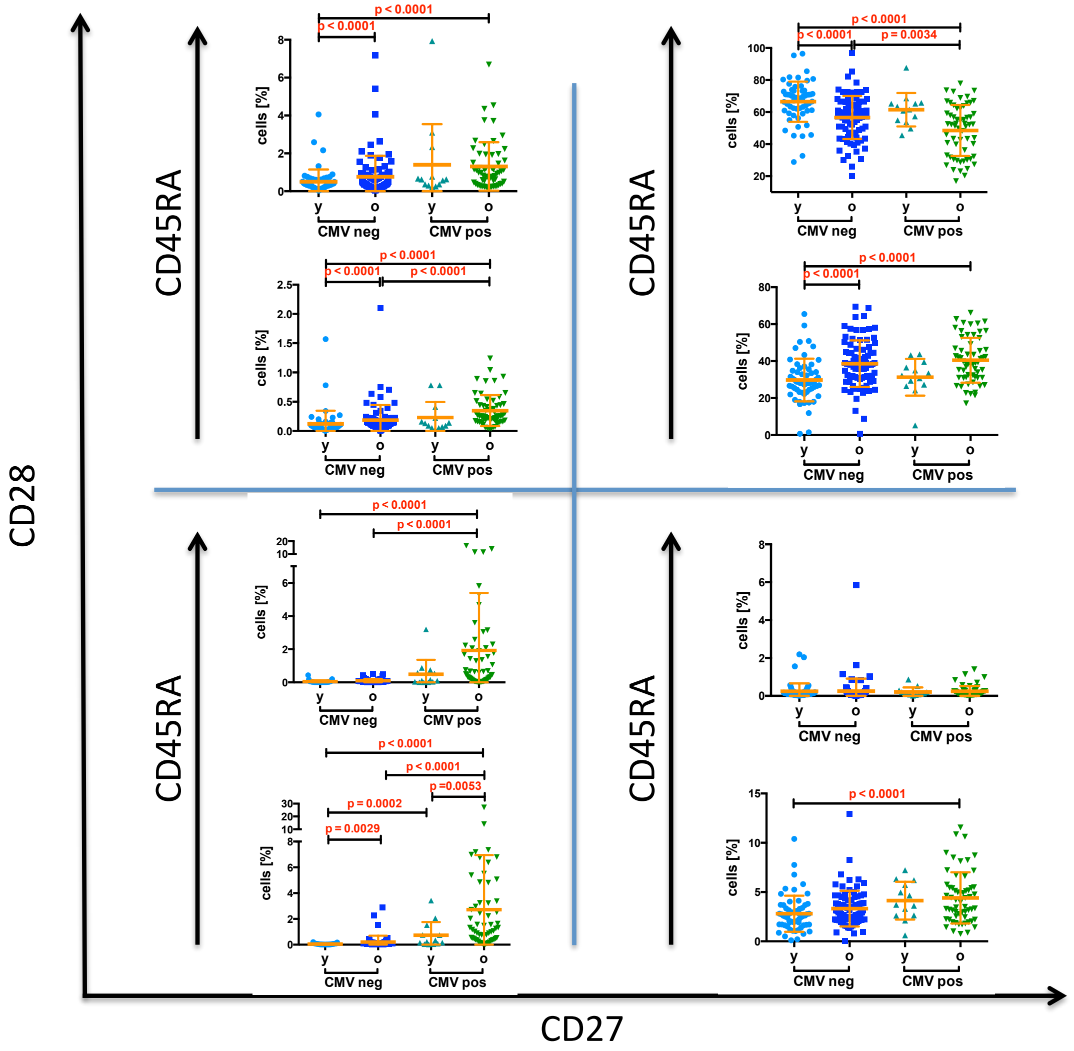

Figure S7: Differentiation phenotypes of the CD4+ αβ T-cells

The differentiation compartments were identified by CD27, CD28, CD45RA and were all CD16 negative in young (y) and old (o) CMV seropositive (CMV pos) and seronegative (CMV neg) individuals.

Mann Whitney U test was used for the statistical comparison of groups. Bonferroni correction adjusted p≤0.0083 as the level of significance.

Figure S8: Gating strategy

Gating strategy – part 1

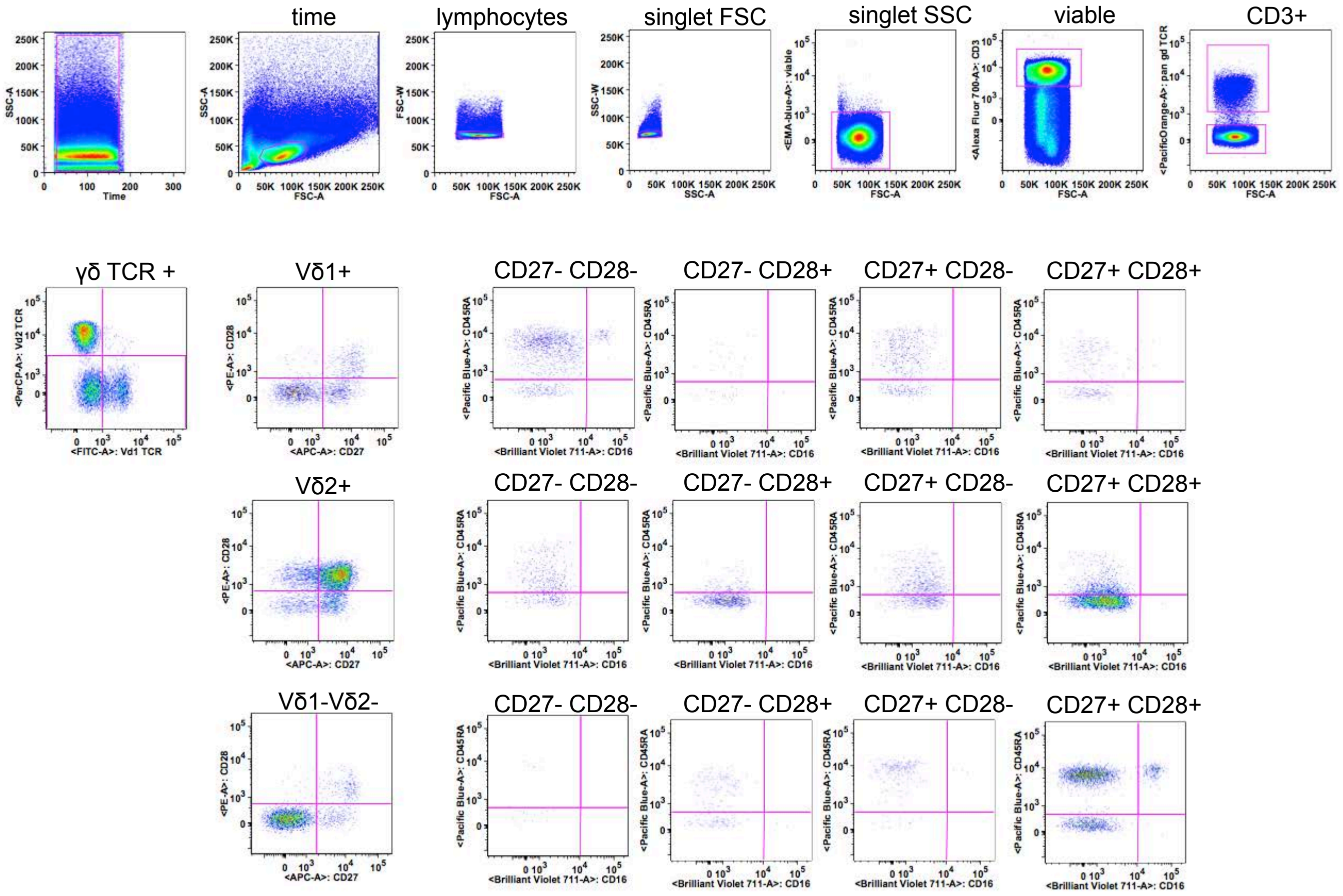

Gating strategy – part 2

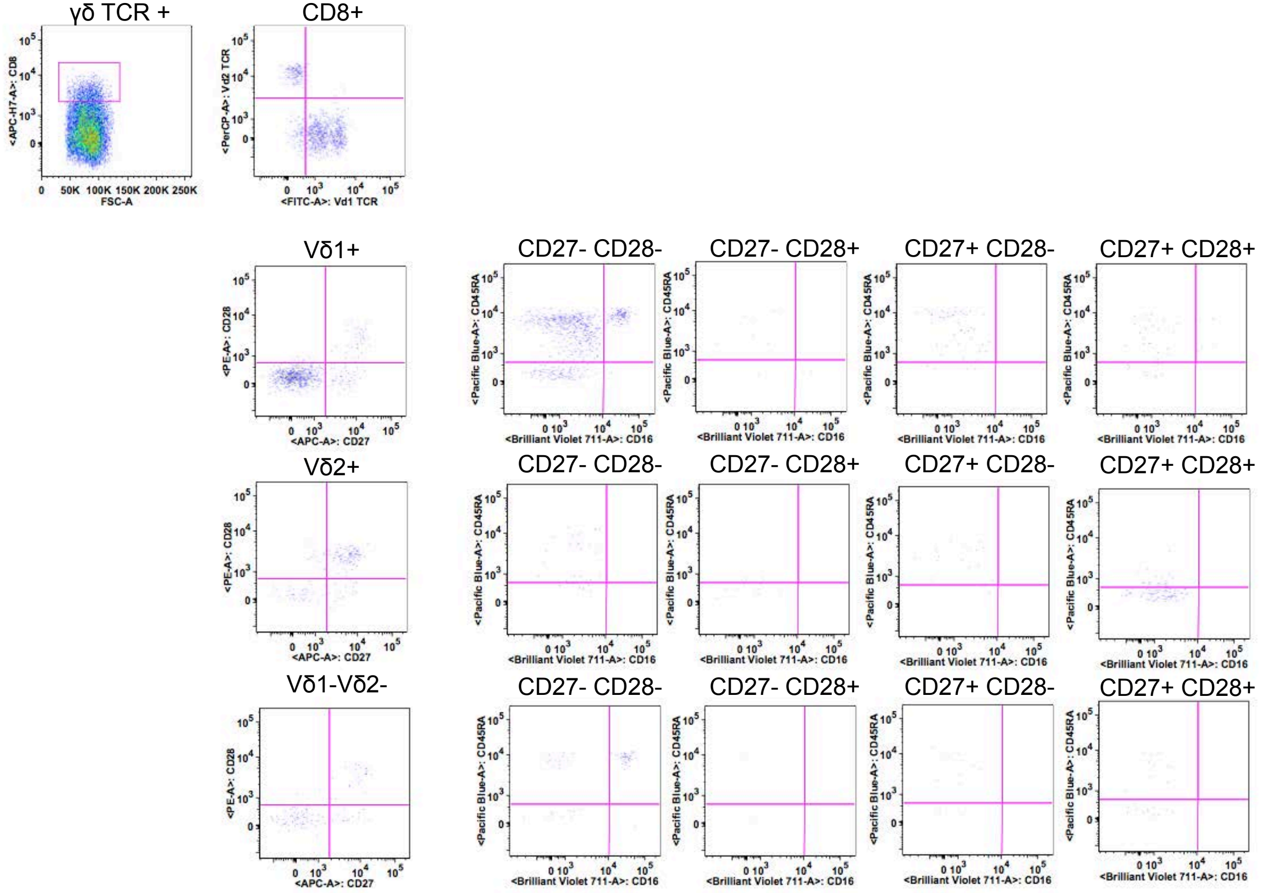

Gating strategy – part 3

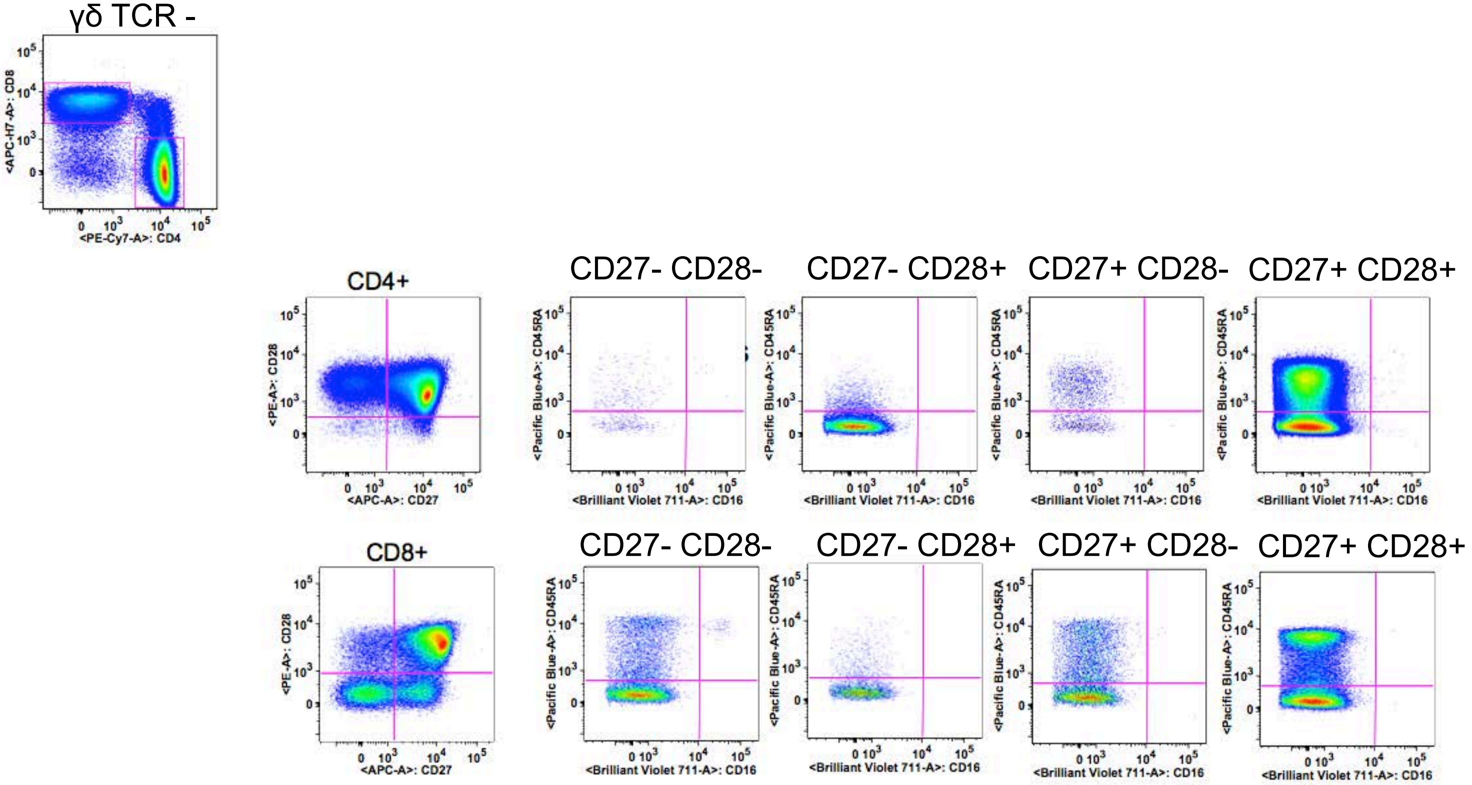

Supplement: Additional file 1: Table S1. — Results of Mann‐Whitney comparisons of the γδ T‐cell compartments and subsets. Table S2. Results of Mann‐Whitney comparisons of the CD8+ γδ T‐cell compartments and subsets. Table S3. Results of Mann‐Whitney comparisons of the αβT‐cell compartments and subsets. Table S4. Results of Mann‐Whitney comparisons of the absolute counts for all T‐cell compartments. Figure S1. Median frequencies and absolute counts of the γδ T‐cell compartments in young and old CMV‐seropositive (CMV+) and seronegative (CMV‐) individuals. Figure S2. Differentiation phenotypes of the Vδ2+ compartment. Figure S3. Differentiation phenotypes of the Vδ1+ compartment. Figure S4. Differentiation phenotypes of the Vδ1‐Vδ2‐compartment. Figure S5. Differentiation phenotypes of the CD8+ γδ T‐cells. Figure S6. Differentiation phenotypes of the CD8+ αβT‐cells. Figure S7. Differentiation phenotypes of the CD4 + αβT‐cells. Figure S8. Gating Strategy T‐cells. (PDF 1365 kb) [file 12979_2015_52_MOESM1_ESM.pdf]
